# Analysis of shared common genetic risk between amyotrophic lateral sclerosis and epilepsy

**DOI:** 10.1016/j.neurobiolaging.2020.04.011

**Published:** 2020-04-18

**Authors:** Dick Schijven, Remi Stevelink, Mark McCormack, Wouter van Rheenen, Jurjen J. Luykx, Bobby P.C. Koeleman, Jan H. Veldink

**Affiliations:** a Department of Neurology and Neurosurgery, UMC Utrecht Brain Center, University Medical Center Utrecht, Utrecht University, Utrecht, the Netherlands; b Department of Psychiatry, UMC Utrecht Brain Center, University Medical Center Utrecht, Utrecht University, Utrecht, the Netherlands; c Department of Translational Neuroscience, UMC Utrecht Brain Center, University Medical Center Utrecht, Utrecht University, Utrecht, the Netherlands; d Center for Molecular Medicine, University Medical Center Utrecht, Utrecht University, Utrecht, the Netherlands; e Department of Molecular and Cellular Therapeutics, The Royal College of Surgeons in Ireland, Dublin, Ireland

**Keywords:** ALS, Epilepsy, Genetic correlation

## Abstract

Because hyper-excitability has been shown to be a shared pathophysiological mechanism, we used the latest and largest genome-wide studies in amyotrophic lateral sclerosis (n = 36,052) and epilepsy (n = 38,349) to determine genetic overlap between these conditions. First, we showed no significant genetic correlation, also when binned on minor allele frequency. Second, we confirmed the absence of polygenic overlap using genomic risk score analysis. Finally, we did not identify pleiotropic variants in meta-analyses of the 2 diseases. Our findings indicate that amyotrophic lateral sclerosis and epilepsy do not share common genetic risk, showing that hyper-excitability in both disorders has distinct origins.

## 1. Introduction

Amyotrophic lateral sclerosis (ALS) is characterized by the progressive degeneration of motor neurons. Cramps and fasciculations are common in ALS patients and indicate axonal hyper-excitability in motor neurons (Kanai et al., 2006). Proposed mechanisms for hyper-excitability in ALS include impaired inhibitory signaling through GABAergic interneurons, astrocytes failing to adequately regulate glutamate levels and extracellular K+ concentrations at the synaptic cleft, and altered ion channel expression in motor neurons (Do-Ha et al., 2018). Ion channel dysfunction has been correlated to muscle weakness and motor neuron degeneration (Geevasinga et al., 2015), while peripheral hyper-excitability and central hyper-excitability are furthermore strong predictors of shorter survival (Kanai et al., 2012; Shimizu et al., 2018). Similarly, in epilepsy, an imbalance of excitatory and inhibitory mechanisms in the brain contributes to seizure pathogenesis. Interestingly, the anti-epileptic drug retigabine has shown acute beneficial effects on peripheral nerve excitability in ALS (Kovalchuk et al., 2018). Of further note, riluzole—a drug used in the treatment of ALS—has been shown to decrease hippocampal epileptiform activity and reduce seizure activity in epilepsy animal models (Diao et al., 2013; Kim et al., 2007).

The beforementioned observations and the availability of recent and large genome-wide association study (GWAS) datasets (The International League Against Epilepsy Consortium on Complex Epilepsies, 2018; van Rheenen et al., 2016), showing that the heritability of each of the 2 diseases is partly explained by common genetic variation, have led us to investigate whether ALS and epilepsy share any common genetic risk.

## 2. Methods

We used individual level data of subjects with European ancestry from recent large GWASs in ALS (N = 12,577 cases; 23,475 controls) (van Rheenen et al., 2016) and epilepsy (N = 14,131 cases; 24,218 controls), including sets of focal (n = 9095 cases) and generalized (n = 3305 cases) epilepsy subtypes (The International League Against Epilepsy Consortium on Complex Epilepsies, 2018). We then performed genetic correlation analyses, genomic risk score (GRS) analyses, and meta-analyses (Supplementary Methods).

## 3. Core data

**Fig. 1.**
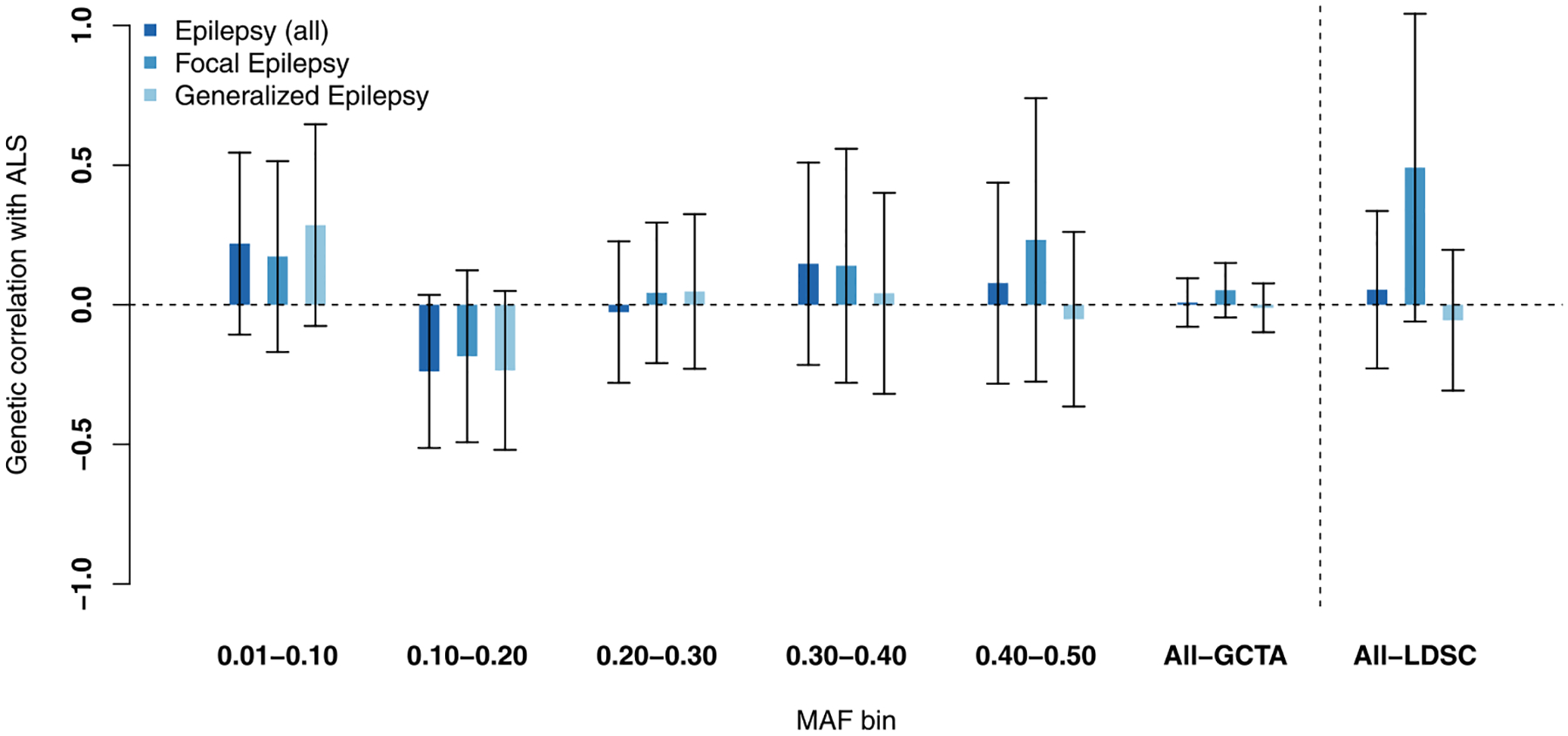
Genetic correlation between ALS and epilepsy. Genetic correlation between ALS and epilepsy (including focal and generalized subtypes) (*y*-axis) was estimated based on all SNPs in the MAF range 0.01–0.5 (GCTA-All) and on SNPs in 5 MAF bins and using GCTA-GREML (*x*-axis). Furthermore, LDSC was used to estimate genetic correlation (All-LDSC). Error bars indicate 95% confidence intervals. Abbreviations: ALS, amyotrophic lateral sclerosis; GCTA, Genome-wide Complex Trait Analysis; GREML, genetic restricted maximum likelihood; LD, linkage disequilibrium; LDSC, linkage disequilibrium score regression; MAF, minor allele frequency; SNP, single-nucleotide polymorphism.

**Fig. 2.**
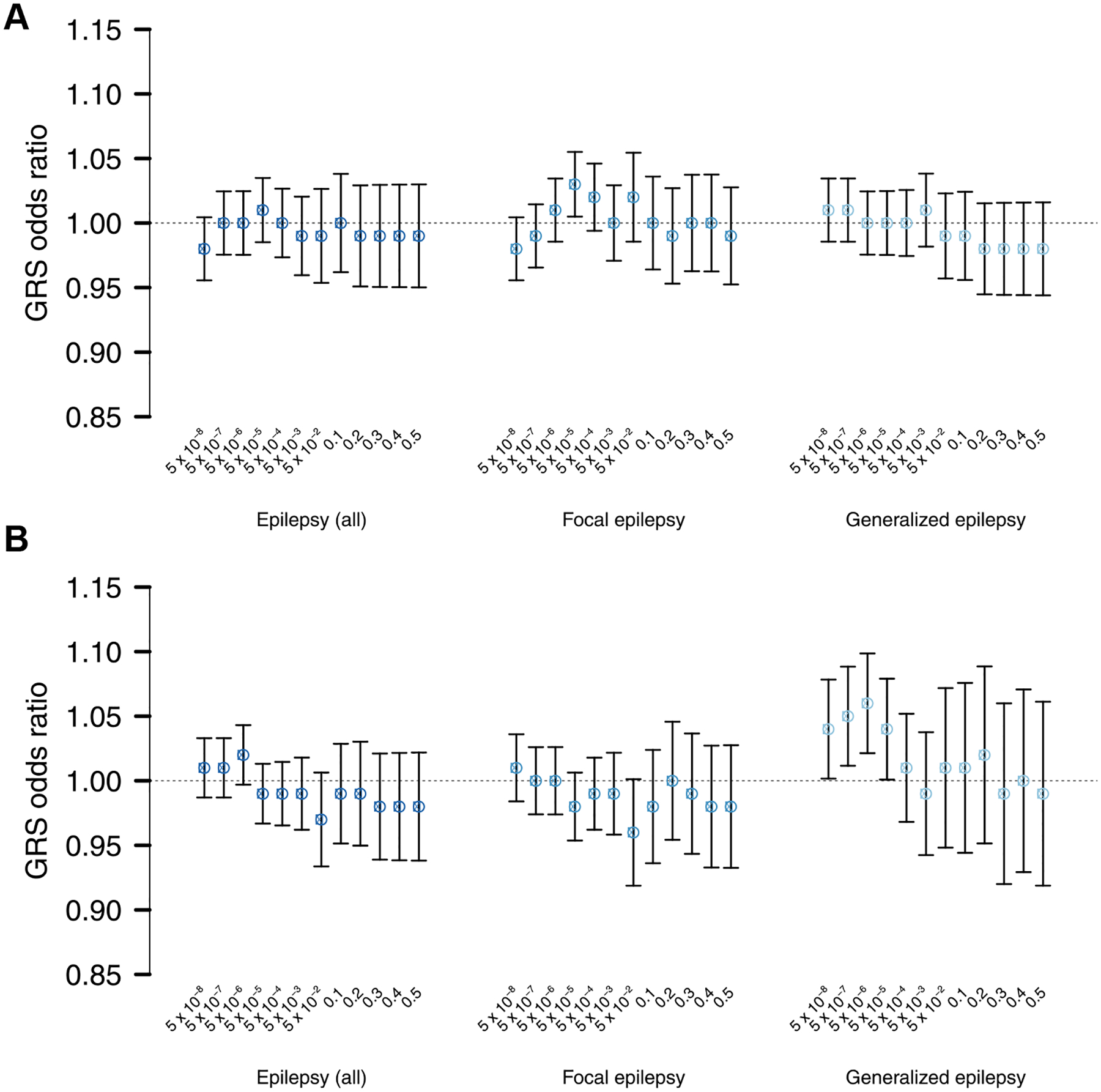
GRS analysis results. Twelve GRSs were calculated per individual in the target dataset and tested for association with the target disease using logistic regression (*x*-axis). (A) Epilepsy GWAS results were used as discovery datasets, and ALS was used as the target dataset. (B) ALS GWAS results were used as the discovery dataset, and epilepsy and subtypes were used as target datasets. Odds ratios reflect the amount by which the odds of ALS (in A) or epilepsy (in B) changes per SD increase in GRS (all GRS were scaled around mean 0 with SD 1). Error bars indicate 95% confidence intervals. The *p*-value threshold for significant GRS association was Bonferroni-corrected for 6 analyses times 12 *P*_T_ (*p* < 6.94 × 10^−4^). Detailed results are shown in Supplementary Table 3. Abbreviations: ALS, amyotrophic lateral sclerosis; GRS, genomic risk score; GWAS, genome-wide association study; SD, standard deviation.

**Fig. 3.**
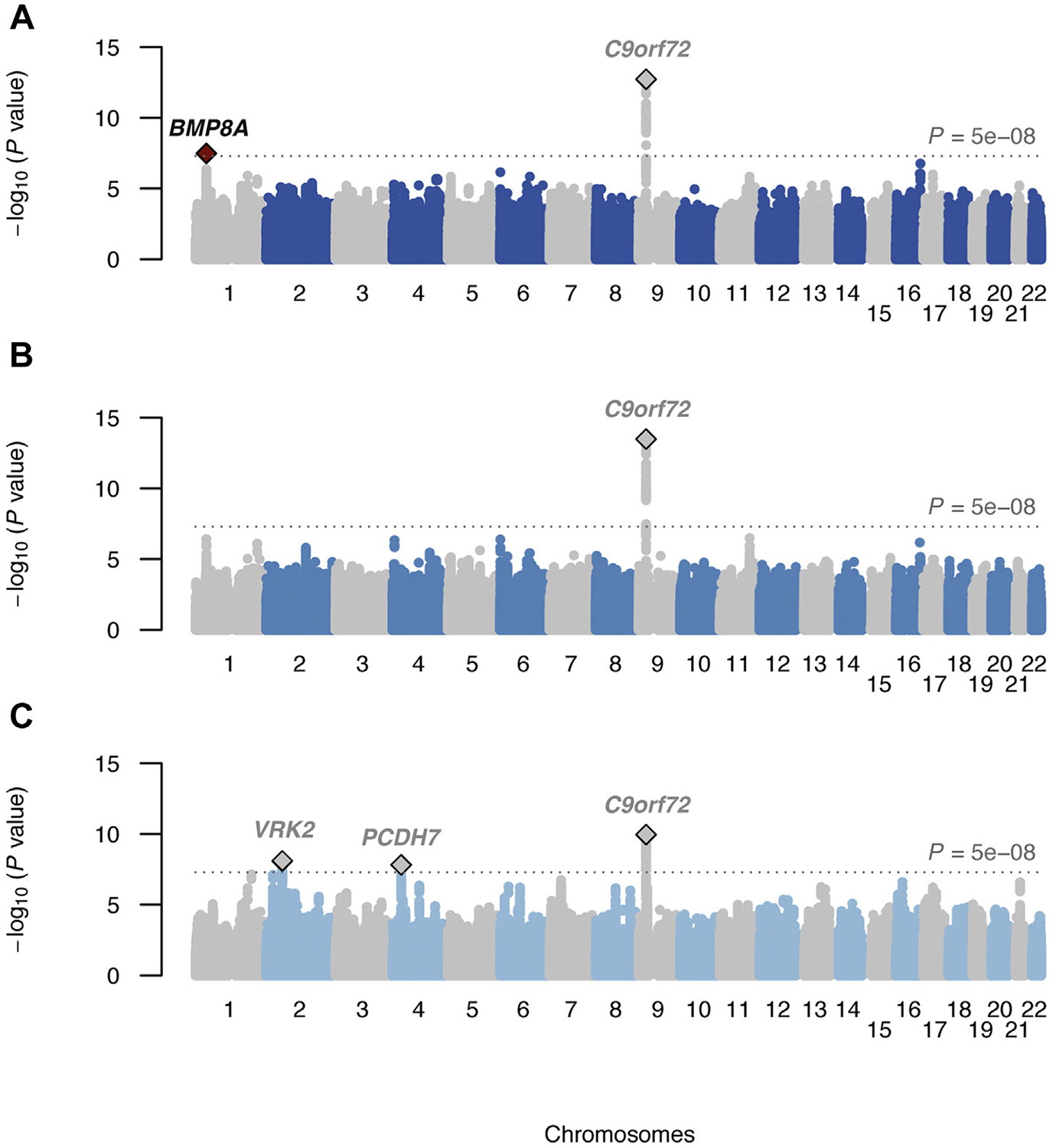
Association results of ALS epilepsy meta-analyses. Manhattan plots show association −log_10_-converted *p*-values (*y*-axis) of meta-analyzed SNPs against their relative position in the genome (*x*-axis). Diamonds indicate lead SNPs of loci reaching genome-wide significance, those marked red indicate pleiotropic loci, and those marked gray indicate loci with a stronger association in the single-phenotype GWAS of ALS or epilepsy compared to the meta-analysis. (A) Result for ALS-epilepsy (41,228 controls; 26,634 cases), with a pleiotropic genome-wide significant association (rs61779331, *p* = 3.2 × 10^−8^, OR = 1.02) near the gene *BMP8A*. (B) Result for ALS-focal epilepsy (41,228 controls; 21,598 cases). (C) Result for ALS-generalized epilepsy (41,228 controls; 15,808 cases). See Supplementary Table 4A for detailed statistics of lead SNPs. Abbreviations: ALS, amyotrophic lateral sclerosis; GWAS, genome-wide association study; OR, odds ratio; SNP, single-nucleotide polymorphism.

We found no significant genetic correlations between ALS and epilepsy, including focal and generalized subtypes (Fig. 1). Furthermore, genetic risk for epilepsy was not associated with case–control status in the ALS dataset (Fig. 2A, Supplementary Table 3A), and similarly ALS GRSs did not associate with epilepsy status (Fig. 2B, Supplementary Table 3B). Inverse-variance weighted meta-analyses of ALS and epilepsy revealed several loci passing the threshold for genome-wide significance (Fig. 3, Supplementary Figs. 2 and 3, Supplementary Table 4). Only the locus near *BMP8A* in the ALS-epilepsy (all subtypes combined) analysis (Fig. 3A) fulfilled criteria for a pleiotropic locus with nominal-significant association *p*-values (*p* < 0.05) in both studies independently (ALS, *p* = 2.3 × 10^−2^; epilepsy, *p* = 8.7 × 10^−8^) and a genome-wide significant *p*-value in meta-analysis (*p* = 3.2 × 10^−8^, odds ratio = 1.02, similar effect directions). This variant, however, was not associated with ALS (*p* = 0.11) in a larger GWAS for which only summary statistics were available (Nicolas et al., 2018).

## 4. Discussion

We have used the latest and largest individual-level genotype datasets for ALS and epilepsy to perform the most comprehensive cross-disorder genome-wide study between these diseases available to date. This has enabled careful control of sample overlap and relatedness between datasets. Also, many tools for cross-disorder analyses have increased power because they use individual-level genotype data instead of summary statistics. Moreover, the genetic correlation estimate between these disorders was not reported in a recent large-scale study on shared polygenic risk in ALS (Bandres Ciga et al., 2019). Overall, we showed with the currently available sample sizes that ALS and epilepsy are not genetically correlated, and that GRS capturing combined effects of common variants in one disease do not explain phenotypic variance in the other disease and that no loci with an effect in both ALS and epilepsy were identified in meta-analyses.

The loci near *BMP8A* and *PTPRK* initially showed evidence for pleiotropy, because of their nominal significant association in both ALS and epilepsy GWAS and stronger statistical association in the meta-analysis and sign-independent meta-analysis, respectively. Nevertheless, the associations of top single-nucleotide polymorphisms (SNPs) in these loci with ALS were weak and did not reach nominal significance in a more recent and larger ALS GWAS meta-analysis (Nicolas et al., 2018), which combined logistic regression association results from summary-level data of van Rheenen et al. (2016) and individual-level data of additional cohorts. Although the meta-analysis framework applied in that larger study could be less powerful compared to a mixed linear model approach with full individual-level data, we were unable prove pleiotropy for these loci. Since only summary statistics of that larger ALS GWAS meta-analysis were available, we were furthermore unable to perform all our analyses including those data. Moreover, the contribution of epilepsy to the pleiotropic signals was large with association *p*-values around the genome-wide significance threshold (The International League Against Epilepsy Consortium on Complex Epilepsies, 2018). These results do not support the notion that these loci are associated with both diseases.

Our results find support in epidemiological data reporting an absence of comorbidity between ALS and epilepsy (Tartaglia et al., 2007), although case reports of co-occurrence in *C9orf72* repeat expansion carriers exist (Capasso et al., 2016; Janssen et al., 2016; van den Ameele et al., 2018). Detailed *C9orf72* repeat expansion status was currently unavailable in our ALS dataset. Although several sodium and potassium channel genes have been associated with epilepsy, and involvement of these channels in the development of cramps in ALS has been proposed (Helbig et al., 2008; Kanai et al., 2006; The International League Against Epilepsy Consortium on Complex Epilepsies, 2018), our results suggest that these clinical observations are not a consequence of shared polygenic architecture. Cramps in ALS might thus be a consequence of disease-specific pathology leading to a hyper-excitable state of motor neurons, for which anti-epileptic drugs can still be effective (Kovalchuk et al., 2018).

We have considered possible limitations of our study. First, we cannot rule out that a study with even larger datasets may possibly yield significant results. However, such a correlation would then be expected to be very small, as we have shown no sign of substantial shared genetic risk with the current large GWAS datasets we have analyzed. The current sample size provides 80% power to detect a genetic correlation of 0.091 at *α* = 0.05 using GCTA-REML (Visscher et al., 2014). Second, rare genetic variation plays a role in both diseases. Although we found non-significant genetic correlation estimates in the lower minor allele frequency (MAF) spectrum of our data, variants with MAF < 0.01 cannot be reliably included in these GWAS analyses. Future GWAS studies employing rare-variant imputation or large-scale sequencing efforts may potentially provide more insight into shared heritability in the rare variant spectrum. Third, phenotypic heterogeneity in ALS and epilepsy might result in a dilution of signal of possible shared genetic underpinnings. Although we have tested genetic correlations with epilepsy subtypes (focal and generalized), a detailed characterization of both ALS and epilepsy patients could identify subgroups that share phenotypic and pathological characteristics that might share a genetic basis. However, at present such phenotypic information is unavailable at sufficiently large sample sizes for cross-disorder analyses.

## Supplementary Material

Supplemental
