# Supplementary material for: Analysis of shared common genetic risk between amyotrophic lateral sclerosis and epilepsy
Source: Neurobiol Aging. Author manuscript; Available in PMC 2021 Jan 21. (PMC7818383; doi:10.1016/j.neurobiolaging.2020.04.011)
Supplement: Supplemental [file NIHMS1658705-supplement-Supplemental.docx]

Supplementary Material

**Analysis of shared common genetic risk between amyotrophic lateral sclerosis and epilepsy**

Dick Schijven, PhD ^a,b,c^, Remi Stevelink, MD ^d^, Mark McCormack, PhD ^d,e^, Wouter van Rheenen, MD, PhD ^a^, Jurjen J. Luykx, MD, PhD ^b^, Bobby P.C. Koeleman, PhD ^d^, Jan H. Veldink, MD, PhD ^a^, on behalf of the Project MinE ALS GWAS Consortium ^f^ and the International League Against Epilepsy Consortium on Complex Epilepsies ^f^

1. Department of Neurology and Neurosurgery, UMC Utrecht Brain Center, University Medical Center Utrecht, Utrecht University, Heidelberglaan 100, 3584 CX, Utrecht, The Netherlands.
2. Department of Psychiatry, UMC Utrecht Brain Center, University Medical Center Utrecht, Utrecht University, Heidelberglaan 100, 3584 CX, Utrecht, the Netherlands
3. Department of Translational Neuroscience, UMC Utrecht Brain Center, University Medical Center Utrecht, Utrecht University, Heidelberglaan 100, 3584 CX, Utrecht, the Netherlands
4. Center for Molecular Medicine, University Medical Center Utrecht, Utrecht University, Heidelberglaan 100, 3584 CX, Utrecht, the Netherlands.
5. Department of Molecular and Cellular Therapeutics, The Royal College of Surgeons in Ireland, 123 St Stephen's Green, Dublin 2, Ireland.
6. A list of consortium members is provided at the end of this document.
7. **Supplementary introduction**

Amyotrophic lateral sclerosis (ALS) is characterized by the progressive degeneration of motor neurons. Cramps and fasciculations are common in ALS patients and indicate axonal hyperexcitability in motor neurons (Kanai et al., 2006). Proposed mechanisms for hyperexcitability in ALS include impaired inhibitory signaling through GABAergic interneurons, astrocytes failing to adequately regulate glutamate levels and extracellular K+ concentrations at the synaptic cleft, and altered ion channel expression in motor neurons (Do-Ha et al., 2018). Ion channel dysfunction has been correlated to muscle weakness and motor neuron degeneration (Geevasinga et al., 2015), while peripheral and central hyperexcitability are furthermore strong predictors of shorter survival (Kanai et al., 2012; Shimizu et al., 2018). Similarly, in epilepsy, an imbalance of excitatory and inhibitory mechanisms in the brain contributes to seizure pathogenesis. Interestingly, the anti-epileptic drug retigabine has shown acute beneficial effects on peripheral nerve excitability in ALS (Kovalchuk et al., 2018). Of further note, riluzole – a drug used in the treatment of ALS – has been shown to decrease hippocampal epileptiform activity and reduce seizure activity in epilepsy animal models (Diao et al., 2013; Kim et al., 2007).

Both ALS and epilepsy have been subject of large genome-wide association studies that revealed parts of their heritabilities being explained by common genetic variation, and that discovered genetic loci associated with the risk for these diseases: For ALS, single-nucleotide polymorphism (SNP)-based heritability has been estimated at 8.5% (van Rheenen et al., 2016). In epilepsy, SNP-based heritability estimates ranged from 9.2% for focal epilepsy to as high as 32.1% for generalized epilepsy (The International League Against Epilepsy Consortium on Complex Epilepsies, 2018). In both ALS and epilepsy, several genetic loci reaching genome-wide significant association have been identified (The International League Against Epilepsy Consortium on Complex Epilepsies, 2018; van Rheenen et al., 2016). Previous research has furthermore established genetic overlap between ALS and other neurological diseases, primarily those in the frontotemporal dementia spectrum (Karch et al., 2018).

The beforementioned clinical and preclinical observations and the availability of recent and large genome-wide association study (GWAS) datasets (The International League Against Epilepsy Consortium on Complex Epilepsies, 2018; van Rheenen et al., 2016), showing that the heritability of each of the two diseases is partly explained by common genetic variation, have led us to investigate whether ALS and epilepsy share any common genetic risk.

1. **Supplementary methods**

**2.1 Study population and data**

We used individual level data of subjects with European ancestry from recent large GWASs in ALS (N = 12,577 cases; 23,475 controls) (van Rheenen et al., 2016) and epilepsy (N = 14,131 cases; 24,218 controls), including sets of focal (n = 9,095 cases) and generalized (n = 3,305 cases) epilepsy subtypes (The International League Against Epilepsy Consortium on Complex Epilepsies, 2018). Contributors to these studies are listed in the Consortium Members section in this document. Written informed consent was provided by all participants and for both studies local institutional review boards at each contributing site approved study protocols (The International League Against Epilepsy Consortium on Complex Epilepsies, 2018; van Rheenen et al., 2016).

**2.2 Genetic correlation analyses**

We merged individual-level data of ALS and epilepsy using PLINK (version v1.90b5.4) (Chang et al., 2015), excluded SNPs with a genotype call rate < 0.99, not present in HapMap3 and/or located in the major histocompatibility complex (MHC) region. Genetic relationship matrices (GRMs) were calculated from SNPs in five minor allele frequency (MAF) bins (MAF 0.01-0.10, 0.10-0.20, 0.20-0.30, 0.30-0.40, and 0.40-0.50, frequencies derived from the European subset of 1000 genomes phase 3, 1KG-EUR, n = 503) and all bins combined (MAF 0.01-0.50) using Genome-wide Complex Trait Analysis (GCTA, version 1.91) (Yang et al., 2011). Because of the stronger contribution of SNPs with low MAF to the heritability of ALS (van Rheenen et al., 2016), we used bivariate genetic restricted maximum likelihood (GREML) to calculate genetic correlation (*r*_g_) per MAF bin and on all bins combined in unrelated individuals (GRM off-diagonal < 0.05) and with the first ten genetic principal components (PCs) derived from GRMs included as covariates.

In support of this, we estimated overall genetic correlation from summary-level data using LD score regression (LDSC) with default settings

(Bulik-Sullivan et al., 2015). LD scores were calculated in 1 centimorgan windows around SNPs present in HapMap3, using 1KG-EUR as a reference dataset. Summary-level data was merged with HapMap3 and combined association statistics of intersecting SNPs between ALS and epilepsy (including focal and generalized subtypes) with 0.05 ≤ MAF ≤ 0.5 were regressed against LD scores.

**2.3 Genomic risk score analyses**

Genomic risk scores (GRS) reflect the sum of discovery GWAS SNP effect alleles, weighted for their effect size, in individuals in a target dataset, and can subsequently be tested for association with a target phenotype. We used the epilepsy mixed linear model (MLM) GWAS results as discovery data and ALS as a target dataset, and vice versa, excluding individuals from the target dataset that were related to individuals used to generate the discovery data (PLINK identity-by-descent PIHAT > 0.10). We excluded variants with a genotype call rate < 0.95 in either the discovery or target dataset. In addition, in the discovery data, we excluded variants other than SNPs, and SNPs with MAF < 0.01, a strand-ambiguous A/T or C/G genotype and those located in genomic regions with a strong or complex LD structure (Supplementary Table 1). LD-independent variants were obtained through clumping of SNPs that intersected with the target dataset, using 1KG-EUR as a reference for LD estimation. Clumping was performed over two rounds: In the first round, SNPs within a 250 kb radius and with an LD R2 > 0.5 relative to a top associated variant were excluded, and in the second round remaining SNPs within a 5000 kb radius and with an LD R2 > 0.2 relative to a top associated variant were removed. Twelve GRS were calculated per individual in the target dataset based on increasing SNP association p-value thresholds (P_T_) in the discovery GWAS (p < 5x10^-8^, p < 5x10^-7^, p < 5x10^-6^, p < 5x10^-5^, p < 5x10^-4^, p < 5x10^-3^, p < 5x10^-2^, p < 0.1, p < 0.2, p < 0.3, p < 0.4 and p < 0.5) using PLINK. GRS were scaled around mean 0 with variance 1 to allow direct comparison of effect sizes between P_T_s. We applied logistic regression, including sex and significant genetic principal components (PCs) (phenotype association p < 0.05/100 = 0.0005) as covariates, to test the association between GRS and disease phenotypes and calculate GRS effect estimates on disease liability.

**2.4 Meta-analyses**

We replicated a previously reported MLM association analysis for ALS using GCTA (van Rheenen et al., 2016), now excluding duplicate and related individuals with the epilepsy data (PLINK PIHAT > 0.10). Inverse-variance weighted fixed effects meta-analyses were performed in METAL for variants with a genotype call rate ≥ 0.95 in MLM GWAS results of ALS and epilepsy (including subtypes) (Willer et al., 2010). Similarly, we performed meta-analyses where we ignored SNP effect direction to discover pleiotropic SNPs with opposing effects in both diseases.

1. **Supplementary results**

We combined ALS and epilepsy GWAS datasets (totaling 74,401 individuals), applied genetic correlation and GRS analyses to assess shared heritability between these diseases, and performed meta-analyses to discover possible pleiotropic loci.

We found no significant genetic correlations using GCTA-GREML between ALS and epilepsy (*r*_g_ = 0.0080 [95% confidence interval -0.079–0.095], p = 0.86), including focal subtype (*r*_g_ = 0.052 [-0.046–0.15], p = 0.29) and generalized subtype (*r*_g_ = -0.011 [-0.099–0.077], p = 0.81), also when the analysis was additionally stratified on MAF (Figure 1, Supplementary Table 2). The absence of genetic correlation with ALS was confirmed using LDSC for all (*r*_g_ = 0.054 [-0.23–0.34], p =0.70), focal (*r*_g_ = 0.49 [-0.060–1.04], p = 0.08), and generalized epilepsy (*r*_g_ = -0.055 [-0.31–0.20], p = 0.67) (Figure 1). Further analysis with LDSC using available summary-level data from a larger GWAS meta-analysis in ALS (Nicolas et al., 2018), revealed highly similar and non-significant genetic correlation estimates (Supplementary Figure 1). Furthermore, GRS capturing genetic risk for epilepsy were not associated with case-control status in the ALS dataset (Figure 2A, Supplementary Table 3A), and similarly ALS GRS did not associate with epilepsy status (Figure 2B, Supplementary Table 3B).

Inverse-variance weighted meta-analyses of ALS and epilepsy revealed several loci passing the threshold for genome-wide significance (Figure 3, Supplementary Figure 2, Supplementary Table 4A). Only the locus near *BMP8A* in the ALS-epilepsy (all subtypes combined) analysis (Figure 3A) fulfilled criteria for a pleiotropic locus with nominal-significant association p-values (p < 0.05) in both studies independently (ALS p = 2.3 x 10^-2^, epilepsy p = 8.7 x 10^-8^) and a genome-wide significant p-value in meta-analysis (p = 3.2 x 10^-8^, OR = 1.02, similar effect directions). This variant, however, was not associated with ALS in a larger GWAS meta-analysis including 20,806 cases and 59,804 controls for which only summary statistics are currently available (p = 0.11, OR [A allele] = 1.03, 95% CI = 0.99-1.06, a corresponding direction of effect compared to the meta-analysis) (Nicolas et al., 2018). For all other loci, the level of association in either ALS or epilepsy alone was higher compared to the combined analysis. In the sign-independent meta-analysis of ALS and generalized epilepsy the signal near *PTPRK* was stronger than both single-trait GWAS (p = 3.5 x 10^-10^, Supplementary Figure 3, Supplementary Table 4B), but again the larger GWAS in ALS did not yield a statistically significant association (p = 0.19, OR [T allele] = 1.02, 95% CI = 0.99-1.05, a corresponding direction of effect compared to the ALS MLM result, but opposing direction of effect compared to the epilepsy MLM result) (Nicolas et al., 2018).

**References**

Bandres Ciga, S., Noyce, A.J., Hemani, G., Nicolas, A., Calvo, A., Mora, G., The ITALSGEN Consortium, The International ALS Genomics Consortium, Tienari, P.J., Stone, D.J., Nalls, M.A., Singleton, A.B., Chiò, A., Traynor, B.J., 2019. Shared polygenic risk and causal inferences in amyotrophic lateral sclerosis. Ann Neurol ana.25431. doi:10.1002/ana.25431

Bulik-Sullivan, B., Finucane, H.K., Anttila, V., Gusev, A., Day, F.R., Loh, P.R., Duncan, L., Perry, J.R., Patterson, N., Robinson, E.B., Daly, M.J., Price, A.L., Neale, B.M., 2015. An atlas of genetic correlations across human diseases and traits. Nat Genet 47, 1236–1241. doi:10.1038/ng.3406

Capasso, M., Anzellotti, F., Di Giacomo, R., Onofrj, M., 2016. Epilepsy and electroencephalographic abnormalities in C9orf72 repeat expansion. Amyotroph Lateral Scler Frontotemporal Degener 18, 140–141. doi:10.1080/21678421.2016.1231825

Chang, C.C., Chow, C.C., Tellier, L.C., Vattikuti, S., Purcell, S.M., Lee, J.J., 2015. Second-generation PLINK: rising to the challenge of larger and richer datasets. GigaScience 4, 7. doi:10.1186/s13742-015-0047-8

Diao, L., Hellier, J.L., Uskert-Newsom, J., Williams, P.A., Staley, K.J., Yee, A.S., 2013. Diphenytoin, riluzole and lidocaine: three sodium channel blockers, with different mechanisms of action, decrease hippocampal epileptiform activity. Neuropharmacology 73, 48–55. doi:10.1016/j.neuropharm.2013.04.057

Do-Ha, D., Buskila, Y., Ooi, L., 2018. Impairments in Motor Neurons, Interneurons and Astrocytes Contribute to Hyperexcitability in ALS: Underlying Mechanisms and Paths to Therapy. Mol. Neurobiol. 55, 1410–1418. doi:10.1007/s12035-017-0392-y

Geevasinga, N., Menon, P., Howells, J., Nicholson, G.A., Kiernan, M.C., Vucic, S., 2015. Axonal ion channel dysfunction in c9orf72 familial amyotrophic lateral sclerosis. JAMA Neurol 72, 49–57. doi:10.1001/jamaneurol.2014.2940

Helbig, I., Scheffer, I.E., Mulley, J.C., Berkovic, S.F., 2008. Navigating the channels and beyond: unravelling the genetics of the epilepsies. Lancet Neurol 7, 231–245. doi:10.1016/S1474-4422(08)70039-5

Janssen, P., Houben, M., Hoff, E., 2016. Photosensitivity in a patient with C9orf72 repeat expansion. Amyotroph Lateral Scler Frontotemporal Degener 17, 266–269. doi:10.3109/21678421.2015.1125503

Kanai, K., Kuwabara, S., Misawa, S., Tamura, N., Ogawara, K., Nakata, M., Sawai, S., Hattori, T., Bostock, H., 2006. Altered axonal excitability properties in amyotrophic lateral sclerosis: impaired potassium channel function related to disease stage. Brain 129, 953–962. doi:10.1093/brain/awl024

Kanai, K., Shibuya, K., Sato, Y., Misawa, S., Nasu, S., Sekiguchi, Y., Mitsuma, S., Isose, S., Fujimaki, Y., Ohmori, S., Koga, S., Kuwabara, S., 2012. Motor axonal excitability properties are strong predictors for survival in amyotrophic lateral sclerosis. J Neurol Neurosurg Psychiatry 83, 734–738. doi:10.1136/jnnp-2011-301782

Karch, C.M., Wen, N., Fan, C.C., Yokoyama, J.S., Kouri, N., Ross, O.A., Höglinger, G., Müller, U., Ferrari, R., Hardy, J., Schellenberg, G.D., Sleiman, P.M., Momeni, P., Hess, C.P., Miller, B.L., Sharma, M., Van Deerlin, V., Smeland, O.B., Andreassen, O.A., Dale, A.M., Desikan, R.S., International Frontotemporal Dementia (FTD)–Genomics Consortium, International Collaboration for Frontotemporal Dementia, Progressive Supranuclear Palsy (PSP) Genetics Consortium, and International Parkinson’s Disease Genomics Consortium, 2018. Selective Genetic Overlap Between Amyotrophic Lateral Sclerosis and Diseases of the Frontotemporal Dementia Spectrum. JAMA Neurol 75, 860–875. doi:10.1001/jamaneurol.2018.0372

Kim, J.-E., Kim, D.-S., Kwak, S.-E., Choi, H.-C., Song, H.-K., Choi, S.-Y., Kwon, O.-S., Kim, Y.-I., Kang, T.-C., 2007. Anti-glutamatergic effect of riluzole: comparison with valproic acid. Neuroscience 147, 136–145. doi:10.1016/j.neuroscience.2007.04.018

Kovalchuk, M.O., Heuberger, J.A.A.C., Sleutjes, B.T.H.M., Ziagkos, D., van den Berg, L.H., Ferguson, T.A., Franssen, H., Groeneveld, G.J., 2018. Acute Effects of Riluzole and Retigabine on Axonal Excitability in Patients With Amyotrophic Lateral Sclerosis: A Randomized, Double-Blind, Placebo-Controlled, Crossover Trial. Clin Pharmacol Ther 9, 65. doi:10.1002/cpt.1096

Nicolas, A., Kenna, K.P., Renton, A.E., Ticozzi, N., Faghri, F., Chia, R., Dominov, J.A., Kenna, B.J., Nalls, M.A., Keagle, P., Rivera, A.M., van Rheenen, W., Murphy, N.A., van Vugt, J.J.F.A., Geiger, J.T., Van der Spek, R.A., Pliner, H.A., Shankaracharya, Smith, B.N., Marangi, G., Topp, S.D., Abramzon, Y., Gkazi, A.S., Eicher, J.D., Kenna, A., ITALSGEN Consortium, Mora, G., Calvo, A., Mazzini, L., Riva, N., Mandrioli, J., Caponnetto, C., Battistini, S., Volanti, P., La Bella, V., Conforti, F.L., Borghero, G., Messina, S., Simone, I.L., Trojsi, F., Salvi, F., Logullo, F.O., D'Alfonso, S., Corrado, L., Capasso, M., Ferrucci, L., Genomic Translation for ALS Care (GTAC) Consortium, Moreno, C. de A.M., Kamalakaran, S., Goldstein, D.B., ALS Sequencing Consortium, Gitler, A.D., Harris, T., Myers, R.M., NYGC ALS Consortium, Phatnani, H., Musunuri, R.L., Evani, U.S., Abhyankar, A., Zody, M.C., Answer ALS Foundation, Kaye, J., Finkbeiner, S., Wyman, S.K., LeNail, A., Lima, L., Fraenkel, E., Svendsen, C.N., Thompson, L.M., Van Eyk, J.E., Berry, J.D., Miller, T.M., Kolb, S.J., Cudkowicz, M., Baxi, E., Clinical Research in ALS and Related Disorders for Therapeutic Development (CReATe) Consortium, Benatar, M., Taylor, J.P., Rampersaud, E., Wu, G., Wuu, J., SLAGEN Consortium, Lauria, G., Verde, F., Fogh, I., Tiloca, C., Comi, G.P., Sorarù, G., Cereda, C., French ALS Consortium, Corcia, P., Laaksovirta, H., Myllykangas, L., Jansson, L., Valori, M., Ealing, J., Hamdalla, H., Rollinson, S., Pickering-Brown, S., Orrell, R.W., Sidle, K.C., Malaspina, A., Hardy, J., Singleton, A.B., Johnson, J.O., Arepalli, S., Sapp, P.C., McKenna-Yasek, D., Polak, M., Asress, S., Al-Sarraj, S., King, A., Troakes, C., Vance, C., de Belleroche, J., Baas, F., Asbroek, ten, A.L.M.A., Munoz-Blanco, J.L., Hernandez, D.G., Ding, J., Gibbs, J.R., Scholz, S.W., Floeter, M.K., Campbell, R.H., Landi, F., Bowser, R., Pulst, S.M., Ravits, J.M., MacGowan, D.J.L., Kirby, J., Pioro, E.P., Pamphlett, R., Broach, J., Gerhard, G., Dunckley, T.L., Brady, C.B., Kowall, N.W., Troncoso, J.C., Le Ber, I., Mouzat, K., Lumbroso, S., Heiman-Patterson, T.D., Kamel, F., Van Den Bosch, L., Baloh, R.H., Strom, T.M., Meitinger, T., Shatunov, A., van Eijk, K.R., de Carvalho, M., Kooyman, M., Middelkoop, B., Moisse, M., McLaughlin, R.L., van Es, M.A., Weber, M., Boylan, K.B., Van Blitterswijk, M., Rademakers, R., Morrison, K.E., Basak, A.N., Mora, J.S., Drory, V.E., Shaw, P.J., Turner, M.R., Talbot, K., Hardiman, O., Williams, K.L., Fifita, J.A., Nicholson, G.A., Blair, I.P., Rouleau, G.A., Esteban-Perez, J., Garcia-Redondo, A., Al-Chalabi, A., Project MinE ALS Sequencing Consortium, Rogaeva, E., Zinman, L., Ostrow, L.W., Maragakis, N.J., Rothstein, J.D., Simmons, Z., Cooper-Knock, J., Brice, A., Goutman, S.A., Feldman, E.L., Gibson, S.B., Taroni, F., Ratti, A., Gellera, C., van Damme, P., Robberecht, W., Fratta, P., Sabatelli, M., Lunetta, C., Ludolph, A.C., Andersen, P.M., Weishaupt, J.H., Camu, W., Trojanowski, J.Q., Van Deerlin, V.M., Brown, R.H., van den Berg, L.H., Veldink, J.H., Harms, M.B., Glass, J.D., Stone, D.J., Tienari, P., Silani, V., Chiò, A., Shaw, C.E., Traynor, B.J., Landers, J.E., 2018. Genome-wide Analyses Identify KIF5A as a Novel ALS Gene. Neuron 97, 1268–1283.e6. doi:10.1016/j.neuron.2018.02.027

Shimizu, T., Bokuda, K., Kimura, H., Kamiyama, T., Nakayama, Y., Kawata, A., Isozaki, E., Ugawa, Y., 2018. Sensory cortex hyperexcitability predicts short survival in amyotrophic lateral sclerosis. Neurology 90, e1578–e1587. doi:10.1212/WNL.0000000000005424

Tartaglia, M.C., Rowe, A., Findlater, K., Orange, J.B., Grace, G., Strong, M.J., 2007. Differentiation Between Primary Lateral Sclerosis and Amyotrophic Lateral Sclerosis. Arch Neurol 64, 232–236. doi:10.1001/archneur.64.2.232

The International League Against Epilepsy Consortium on Complex Epilepsies, 2018. Genome-wide mega-analysis identifies 16 loci and highlights diverse biological mechanisms in the common epilepsies. Nat Commun 9, 5269. doi:10.1038/s41467-018-07524-z

van den Ameele, J., Jedlickova, I., Pristoupilova, A., Sieben, A., Van Mossevelde, S., Ceuterick-de Groote, C., Hůlková, H., Matej, R., Meurs, A., Van Broeckhoven, C., Berkovic, S.F., Santens, P., Kmoch, S., Dermaut, B., 2018. Teenage-onset progressive myoclonic epilepsy due to a familial C9orf72 repeat expansion. Neurology 90, e658–e663. doi:10.1212/WNL.0000000000004999

van Rheenen, W., Shatunov, A., Dekker, A.M., McLaughlin, R.L., Diekstra, F.P., Pulit, S.L., van der Spek, R.A.A., Võsa, U., de Jong, S., Robinson, M.R., Yang, J., Fogh, I., van Doormaal, P.T., Tazelaar, G.H.P., Koppers, M., Blokhuis, A.M., Sproviero, W., Jones, A.R., Kenna, K.P., van Eijk, K.R., Harschnitz, O., Schellevis, R.D., Brands, W.J., Medic, J., Menelaou, A., Vajda, A., Ticozzi, N., Lin, K., Rogelj, B., Vrabec, K., Ravnik-Glavač, M., Koritnik, B., Zidar, J., Leonardis, L., Grošelj, L.D., Millecamps, S., Salachas, F., Meininger, V., de Carvalho, M., Pinto, S., Mora, J.S., Rojas-García, R., Polak, M., Chandran, S., Colville, S., Swingler, R., Morrison, K.E., Shaw, P.J., Hardy, J., Orrell, R.W., Pittman, A., Sidle, K., Fratta, P., Malaspina, A., Topp, S., Petri, S., Abdulla, S., Drepper, C., Sendtner, M., Meyer, T., Ophoff, R.A., Staats, K.A., Wiedau-Pazos, M., Lomen-Hoerth, C., Van Deerlin, V.M., Trojanowski, J.Q., Elman, L., McCluskey, L., Basak, A.N., Tunca, C., Hamzeiy, H., Parman, Y., Meitinger, T., Lichtner, P., Radivojkov-Blagojevic, M., Andres, C.R., Maurel, C., Bensimon, G., Landwehrmeyer, B., Brice, A., Payan, C.A.M., Saker-Delye, S., Dürr, A., Wood, N.W., Tittmann, L., Lieb, W., Franke, A., Rietschel, M., Cichon, S., Nothen, M.M., Amouyel, P., Tzourio, C., Dartigues, J.-F., Uitterlinden, A.G., Rivadeneira, F., Estrada, K., Hofman, A., Curtis, C., Blauw, H.M., van der Kooi, A.J., de Visser, M., Goris, A., Weber, M., Shaw, C.E., Smith, B.N., Pansarasa, O., Cereda, C., Del Bo, R., Comi, G.P., D'Alfonso, S., Bertolin, C., Sorarù, G., Mazzini, L., Pensato, V., Gellera, C., Tiloca, C., Ratti, A., Calvo, A., Moglia, C., Brunetti, M., Arcuti, S., Capozzo, R., Zecca, C., Lunetta, C., Penco, S., Riva, N., Padovani, A., Filosto, M., Muller, B., Stuit, R.J., PARALS Registry, SLALOM Group, SLAP Registry, FALS Sequencing Consortium, SLAGEN Consortium, NNIPPS Study Group, Blair, I., Zhang, K., McCann, E.P., Fifita, J.A., Nicholson, G.A., Rowe, D.B., Pamphlett, R., Kiernan, M.C., Grosskreutz, J., Witte, O.W., Ringer, T., Prell, T., Stubendorff, B., Kurth, I., Hübner, C.A., Leigh, P.N., Casale, F., Chiò, A., Beghi, E., Pupillo, E., Tortelli, R., Logroscino, G., Powell, J., Ludolph, A.C., Weishaupt, J.H., Robberecht, W., van Damme, P., Franke, L., Pers, T.H., Brown, R.H., Glass, J.D., Landers, J.E., Hardiman, O., Andersen, P.M., Corcia, P., Vourc'h, P., Silani, V., Wray, N.R., Visscher, P.M., de Bakker, P.I.W., van Es, M.A., Pasterkamp, R.J., Lewis, C.M., Breen, G., Al-Chalabi, A., van den Berg, L.H., Veldink, J.H., 2016. Genome-wide association analyses identify new risk variants and the genetic architecture of amyotrophic lateral sclerosis. Nat Genet 48, 1043–1048. doi:10.1038/ng.3622

Visscher, P.M., Hemani, G., Vinkhuyzen, A.A.E., Chen, G.-B., Lee, S.H., Wray, N.R., Goddard, M.E., Yang, J., 2014. Statistical power to detect genetic (co)variance of complex traits using SNP data in unrelated samples. PLoS Genet. 10, e1004269. doi:10.1371/journal.pgen.1004269

Willer, C.J., Li, Y., Abecasis, G.R., 2010. METAL: fast and efficient meta-analysis of genomewide association scans. Bioinformatics 26, 2190–2191. doi:10.1093/bioinformatics/btq340

Yang, J., Lee, S.H., Goddard, M.E., Visscher, P.M., 2011. GCTA: A Tool for Genome-wide Complex Trait Analysis. Am J Hum Genet 88, 76–82. doi:10.1016/j.ajhg.2010.11.011

**Supplementary Figures**

**Supplementary Figure 1 LDSC genetic correlation estimates between ALS and epilepsy using ALS GWAS data from van Rheenen *et al.* and Nicolas *et al.*** Genetic correlation between ALS and epilepsy (including focal and generalized subtypes) (y-axis) was estimated using LDSC using data from van Rheenen *et al.* (All-LDSC), and Nicolas *et al.* (All-LDSC Nicolas)). Error bars indicate 95% confidence intervals.

**Supplementary Figure 2 Observed versus expected association p-values in ALS-epilepsy meta-analyses**. Quantile-quantile plots for meta-analyses of **A)** ALS-all epilepsy, **B)** ALS-focal epilepsy, and **C)** ALS-generalized epilepsy. Inflation factor lambda (λ_GC_), and lambda scaled for a study with 1,000 cases and 1,000 controls (λ_GC1000_) are shown in each plot.

**Supplementary Figure 3 Sign-independent meta-analysis results.** Manhattan plots show association -log_10_-converted p-values (y-axis) of meta-analyzed SNPs against their relative position on the genome (x-axis). Diamonds indicate lead SNPs of loci reaching genome-wide significance, those marked red indicate pleiotropic loci, those marked gray indicate loci with a stronger association in the single-phenotype GWAS of ALS or epilepsy compared to the meta-analysis. **A)** Sign-independent meta-analysis of ALS-epilepsy (41,228 controls; 26,634 cases) where the same pleiotropic locus as reported in in Figure 3A was discovered. **B)** Sign-independent meta-analysis of ALS-focal epilepsy (41,228 controls; 21,598 cases). **C)** Sign-independent meta-analysis of ALS-generalized epilepsy (41,228 controls; 15,808 cases) with a pleiotropic association (opposing effect directions between ALS and epilepsy, rs13219424, p = 3.5 x 10^-10^, OR = 1.02) near the gene *PTPRK*. See Supplementary Table 4 for detailed statistics of lead SNPs.

**Supplementary Tables**

**Supplementary Table 1 Genomic regions of strong LD excluded in genomic risk score calculation**

| **Chromosome** | **Basepair start** | **Basepair end** |
| --- | --- | --- |
| **1** | 48000000 | 52000000 |
| **2** | 86000000 | 100500000 |
|  | 183000000 | 190000000 |
| **3** | 47500000 | 50000000 |
|  | 83500000 | 87000000 |
| **5** | 44500000 | 50500000 |
|  | 129000000 | 132000000 |
| **6** | 25500000 | 33500000 |
|  | 57000000 | 64000000 |
|  | 140000000 | 142500000 |
| **7** | 55000000 | 66000000 |
| **8** | 8000000 | 12000000 |
|  | 43000000 | 50000000 |
|  | 8135000 | 12000000 |
|  | 112000000 | 115000000 |
| **10** | 37000000 | 43000000 |
| **11** | 87500000 | 90500000 |
| **12** | 33000000 | 40000000 |
| **17** | 40900000 | 45000000 |
| **20** | 32000000 | 34500000 |

Base pair positions refer to human genome build hg19/GRCh37.

**Supplementary Table 2 Results for GREML analysis of ALS and epilepsy subtypes**

|  | ***________________h*^2^ ALS________________** | | ***______________h*^2^ Epilepsy______________** | |  |  |
| --- | --- | --- | --- | --- | --- | --- |
| **MAF bin** | **Observed scale (SE)** | **Liability scale (SE)** | **Observed scale (SE)** | **Liability scale (SE)** | ***cov*_g_ (SE)** | ***r*_g_ (SE)** |
| **ALS-All epilepsy** |  |  |  |  |  |  |
| All | 0.024131 (0.001927) | 0.051265 (0.004033) | 0.057662 (0.002737) | 0.125524 (0.005635) | 0.000298 (0.001658) | 0.007996 (0.044454) |
| 0.01-0.10 | 0.004253 (0.001212) | 0.009036 (0.002573) | 0.008871 (0.001635) | 0.019312 (0.003547) | 0.001345 (0.001001) | 0.218974 (0.166300) |
| 0.10-0.20 | 0.005659 (0.001499) | 0.012021 (0.003180) | 0.014908 (0.002062) | 0.032454 (0.004461) | -0.002193 (0.001252) | -0.238750 (0.139819) |
| 0.20-0.30 | 0.007977 (0.001525) | 0.016946 (0.003232) | 0.011688 (0.002048) | 0.025444 (0.004440) | -0.000255 (0.001249) | -0.026419 (0.129395) |
| 0.30-0.40 | 0.003182 (0.001395) | 0.006759 (0.002963) | 0.013188 (0.002023) | 0.028709 (0.004381) | 0.000951 (0.001185) | 0.146786 (0.184884) |
| 0.40-0.50 | 0.003510 (0.001231) | 0.007456 (0.002614) | 0.009251 (0.001767) | 0.020138 (0.003835) | 0.000442 (0.001045) | 0.077637 (0.183738) |
| **ALS-Focal epilepsy** |  |  |  |  |  |  |
| All | 0.024157 (0.001928) | 0.051318 (0.004033) | 0.047059 (0.002831) | 0.107541 (0.006217) | 0.001755 (0.001681) | 0.052051 (0.049853) |
| 0.01-0.10 | 0.004266 (0.001212) | 0.009063 (0.002573) | 0.008475 (0.001749) | 0.019369 (0.003985) | 0.001038 (0.001035) | 0.172609 (0.174416) |
| 0.10-0.20 | 0.005642 (0.001499) | 0.011985 (0.003180) | 0.012106 (0.002150) | 0.027668 (0.004892) | -0.001526 (0.001279) | -0.184597 (0.157118) |
| 0.20-0.30 | 0.007999 (0.001526) | 0.016993 (0.003232) | 0.012514 (0.002166) | 0.028599 (0.004926) | 0.000427 (0.001286) | 0.042652 (0.128468) |
| 0.30-0.40 | 0.003203 (0.001396) | 0.006805 (0.002964) | 0.009886 (0.002071) | 0.022592 (0.004718) | 0.000785 (0.001198) | 0.139536 (0.213927) |
| 0.40-0.50 | 0.003486 (0.001231) | 0.007405 (0.002612) | 0.004861 (0.001797) | 0.011110 (0.004102) | 0.000956 (0.001048) | 0.232349 (0.258904) |
| **ALS-Generalized epilepsy** |  |  |  |  |  |  |
| All | 0.024125 (0.001927) | 0.051253 (0.004033) | 0.044636 (0.002124) | 0.271195 (0.011908) | -0.000362 (0.001466) | -0.011025 (0.044666) |
| 0.01-0.10 | 0.004251 (0.001212) | 0.009030 (0.002572) | 0.005642 (0.001240) | 0.034281 (0.007509) | 0.001397 (0.000875) | 0.285209 (0.184363) |
| 0.10-0.20 | 0.005665 (0.001499) | 0.012035 (0.003180) | 0.010549 (0.001591) | 0.064102 (0.009598) | -0.001818 (0.001097) | -0.235176 (0.145131) |
| 0.20-0.30 | 0.008021 (0.001526) | 0.017041 (0.003233) | 0.007457 (0.001574) | 0.045313 (0.009529) | 0.000368 (0.001093) | 0.047524 (0.141351) |
| 0.30-0.40 | 0.003088 (0.001394) | 0.006560 (0.002961) | 0.010714 (0.001590) | 0.065101 (0.009582) | 0.000236 (0.001055) | 0.041081 (0.183603) |
| 0.40-0.50 | 0.003537 (0.001231) | 0.007515 (0.002614) | 0.009621 (0.001395) | 0.058463 (0.008410) | -0.000302 (0.000930) | -0.051770 (0.159512) |

Liability-scale *h*^2^ in ALS was calculated using a prevalence of 0.0025, Liability-scale *h*^2^ in epilepsy was calculated using a prevalence of 0.006 for all epilepsy, 0.003 for focal epilepsy, and 0.002 for generalized epilepsy. MAF, minor allele frequency; *h*^2^, heritability; *cov*_g_, genetic covariance; *r*_g_, genetic correlation; SE, standard error.

**Supplementary Table 3 Genomic risk score analysis results**

**A**

|  | **Discovery dataset** | | | | | | | | | | | | | | |
| --- | --- | --- | --- | --- | --- | --- | --- | --- | --- | --- | --- | --- | --- | --- | --- |
|  | **All Epilepsy** | | | | | **Focal Epilepsy** | | | | | **Generalized Epilepsy** | | | | |
| **P_T_** | **SNPs** | **OR** | **se** | **Delta R^2^** | **p** | **SNPs** | **OR** | **se** | **Delta R^2^** | **p** | **SNPs** | **OR** | **se** | **Delta R^2^** | **p** |
| 5 x 10^-8^ | 4 | 0.98 | 1.2 x 10^-2^ | 1.4 x 10^-4^ | 0.06 | 1 | 0.98 | 1.2 x 10^-2^ | 6.7 x 10^-5^ | 0.20 | 8 | 1.01 | 1.2 x 10^-2^ | 1.1 x 10^-5^ | 0.60 |
| 5 x 10^-7^ | 16 | 1.00 | 1.3 x 10^-2^ | 1.1 x 10^-6^ | 0.87 | 3 | 0.99 | 1.2 x 10^-2^ | 2.5 x 10^-5^ | 0.43 | 20 | 1.01 | 1.2 x 10^-2^ | 3.3 x 10^-5^ | 0.37 |
| 5 x 10^-6^ | 55 | 1.00 | 1.3 x 10^-2^ | 1.8 x 10^-6^ | 0.83 | 30 | 1.01 | 1.2 x 10^-2^ | 3.8 x 10^-5^ | 0.33 | 46 | 1.00 | 1.2 x 10^-2^ | 2.6 x 10^-6^ | 0.80 |
| 5 x 10^-5^ | 206 | 1.01 | 1.3 x 10^-2^ | 1.1 x 10^-5^ | 0.60 | 156 | **1.03** | **1.3 x 10^-2^** | **2.4 x 10^-4^** | **0.02** | 180 | 1.00 | 1.3 x 10^-2^ | 2.4 x 10^-6^ | 0.81 |
| 5 x 10^-4^ | 1,092 | 1.00 | 1.4 x 10^-2^ | 3.1 x 10^-7^ | 0.93 | 847 | 1.02 | 1.3 x 10^-2^ | 7.1 x 10^-5^ | 0.19 | 799 | 1.00 | 1.3 x 10^-2^ | 5.3 x 10^-6^ | 0.72 |
| 5 x 10^-3^ | 5,244 | 0.99 | 1.6 x 10^-2^ | 1.3 x 10^-5^ | 0.57 | 4517 | 1.00 | 1.5 x 10^-2^ | 4.4 x 10^-6^ | 0.74 | 4225 | 1.01 | 1.4 x 10^-2^ | 2.6 x 10^-5^ | 0.42 |
| 5 x 10^-2^ | 24,620 | 0.99 | 1.9 x 10^-2^ | 3.7 x 10^-6^ | 0.76 | 22507 | 1.02 | 1.8 x 10^-2^ | 4.5 x 10^-5^ | 0.29 | 21052 | 0.99 | 1.7 x 10^-2^ | 6.0 x 10^-6^ | 0.70 |
| 0.1 | 38,975 | 1.00 | 1.9 x 10^-2^ | 6.3 x 10^-7^ | 0.90 | 36594 | 1.00 | 1.8 x 10^-2^ | 5.7 x 10^-7^ | 0.91 | 34571 | 0.99 | 1.7 x 10^-2^ | 3.4 x 10^-6^ | 0.77 |
| 0.2 | 58,330 | 0.99 | 2.0 x 10^-2^ | 1.1 x 10^-5^ | 0.61 | 55623 | 0.99 | 1.9 x 10^-2^ | 7.9 x 10^-6^ | 0.66 | 53535 | 0.98 | 1.8 x 10^-2^ | 3.3 x 10^-5^ | 0.37 |
| 0.3 | 72,518 | 0.99 | 2.0 x 10^-2^ | 7.7 x 10^-6^ | 0.66 | 69907 | 1.00 | 1.9 x 10^-2^ | 1.6 x 10^-6^ | 0.84 | 68111 | 0.98 | 1.8 x 10^-2^ | 4.8 x 10^-5^ | 0.28 |
| 0.4 | 83,813 | 0.99 | 2.0 x 10^-2^ | 6.9 x 10^-6^ | 0.68 | 81115 | 1.00 | 1.9 x 10^-2^ | 3.7 x 10^-7^ | 0.92 | 79566 | 0.98 | 1.8 x 10^-2^ | 4.1 x 10^-5^ | 0.32 |
| 0.5 | 92,480 | 0.99 | 2.0 x 10^-2^ | 8.8 x 10^-6^ | 0.64 | 89911 | 0.99 | 1.9 x 10^-2^ | 2.9 x 10^-6^ | 0.79 | 88794 | 0.98 | 1.8 x 10^-2^ | 3.0 x 10^-5^ | 0.39 |

**B**

|  |  | **Target dataset** | | | | | | | | | | | |
| --- | --- | --- | --- | --- | --- | --- | --- | --- | --- | --- | --- | --- | --- |
|  |  | **All Epilepsy** | | | | **Focal Epilepsy** | | | | **Generalized Epilepsy** | | | |
| **P_T_** | **SNPs** | **OR** | **se** | **Delta R^2^** | **p** | **OR** | **se** | **Delta R^2^** | **p** | **OR** | **se** | **Delta R^2^** | **p** |
| 5 x 10^-8^ | 4 | 1.01 | 1.2 x 10^-2^ | 5.6 x 10^-5^ | 0.23 | 1.01 | 1.3 x 10^-2^ | 7.7 x 10^-6^ | 0.69 | 1.04 | 2.0 x 10^-2^ | 2.9 x 10^-2^ | 0.05 |
| 5 x 10^-7^ | 8 | 1.01 | 1.2 x 10^-2^ | 1.3 x 10^-5^ | 0.57 | 1.00 | 1.3 x 10^-2^ | 5.2 x 10^-6^ | 0.74 | **1.05** | **2.0 x 10^-2^** | **4.2 x 10^-2^** | **0.02** |
| 5 x 10^-6^ | 17 | 1.02 | 1.2 x 10^-2^ | 6.6 x 10^-5^ | 0.19 | 1.00 | 1.3 x 10^-2^ | 1.0 x 10^-6^ | 0.89 | **1.06** | **2.0 x 10^-2^** | **7.2 x 10^-2^** | **0.002** |
| 5 x 10^-5^ | 86 | 0.99 | 1.2 x 10^-2^ | 7.6 x 10^-6^ | 0.66 | 0.98 | 1.3 x 10^-2^ | 8.4 x 10^-5^ | 0.19 | 1.04 | 2.0 x 10^-2^ | 2.9 x 10^-2^ | 0.05 |
| 5 x 10^-4^ | 483 | 0.99 | 1.3 x 10^-2^ | 1.6 x 10^-5^ | 0.53 | 0.99 | 1.4 x 10^-2^ | 2.6 x 10^-5^ | 0.46 | 1.01 | 2.1 x 10^-2^ | 5.4 x 10^-4^ | 0.79 |
| 5 x 10^-3^ | 2,836 | 0.99 | 1.4 x 10^-2^ | 1.4 x 10^-5^ | 0.56 | 0.99 | 1.6 x 10^-2^ | 3.2 x 10^-5^ | 0.42 | 0.99 | 2.4 x 10^-2^ | 7.5 x 10^-4^ | 0.75 |
| 5 x 10^-2^ | 17,125 | 0.97 | 1.9 x 10^-2^ | 7.9 x 10^-5^ | 0.16 | 0.96 | 2.1 x 10^-2^ | 1.6 x 10^-4^ | 0.07 | 1.01 | 3.2 x 10^-2^ | 7.0 x 10^-4^ | 0.76 |
| 0.1 | 28,319 | 0.99 | 2.0 x 10^-2^ | 2.1 x 10^-5^ | 0.46 | 0.98 | 2.2 x 10^-2^ | 4.3 x 10^-5^ | 0.35 | 1.01 | 3.4 x 10^-2^ | 5.7 x 10^-4^ | 0.78 |
| 0.2 | 46,467 | 0.99 | 2.1 x 10^-2^ | 2.8 x 10^-6^ | 0.79 | 1.00 | 2.3 x 10^-2^ | 1.7 x 10^-6^ | 0.85 | 1.02 | 3.5 x 10^-2^ | 1.9 x 10^-3^ | 0.62 |
| 0.3 | 60,997 | 0.98 | 2.1 x 10^-2^ | 2.9 x 10^-5^ | 0.39 | 0.99 | 2.4 x 10^-2^ | 1.0 x 10^-5^ | 0.65 | 0.99 | 3.6 x 10^-2^ | 2.7 x 10^-4^ | 0.85 |
| 0.4 | 73,344 | 0.98 | 2.1 x 10^-2^ | 3.7 x 10^-5^ | 0.33 | 0.98 | 2.4 x 10^-2^ | 2.5 x 10^-5^ | 0.47 | 1.00 | 3.6 x 10^-2^ | 3.0 x 10^-5^ | 0.95 |
| 0.5 | 83,607 | 0.98 | 2.1 x 10^-2^ | 4.4 x 10^-5^ | 0.29 | 0.98 | 2.4 x 10^-2^ | 3.5 x 10^-5^ | 0.40 | 0.99 | 3.6 x 10^-2^ | 1.9 x 10^-4^ | 0.88 |

**A** Results using epilepsy (including subtypes) as discovery datasets and ALS as a target dataset. Explained variance in ALS (case-control status) by epilepsy GRSs was tested using logistic regression, including sex and significant principal components (p < 0.05/100 = 5x10^-4^; PCs 1, 2, 3, 5, 12, 17, 18, 19, 21, 26, 28, 29, 33, 43, 45, 46, 47, 53, 65, 70, 75, 96) as covariates.

**B** Results using ALS as discovery dataset and epilepsy (including subtypes) as a target dataset. Explained variance in epilepsy (case-control status) by ALS GRSs was tested using logistic regression, including sex and significant principal components (p < 0.05/100 = 5x10^-4^; all epilepsy: PCs 1, 2, 3, 6, 7, 22, 24, 31, 95; focal epilepsy: PCs 1, 2, 3, 6, 7, 8, 22, 24, 31; generalized epilepsy: PCs1, 2, 3, 7, 8, 9, 24, 44, 66, 95) as covariates.

GRSs were scaled around mean 0 with variance 1. OR reflects the odds for disease per one unit (one SD) increase of the GRS at the respective P_T_.

Table columns: P_T_, p-value threshold of SNP association in discovery data; SNPs, number of SNPs in P_T_ (in B this is the maximum number of SNPs due to missing SNP genotypes; OR, odds ratio of the GRS; se, standard error of the effect estimate of the GRS; Delta R^2^, difference in explained variance (Nagelkerke R^2^) compared to baseline model without GRS; p, p-value of the GRS in the model.

Results in bold show GRS effects with p < 0.05, although none survived multiple-testing correction (Bonferroni-correction for four traits times twelve P_T_ included, p < 0.001).

**Supplementary Table 4 Meta-analysis association results of lead SNPs**

| **A. Normal meta-analysis** | | | | | | | | | | | | | | | | |
| --- | --- | --- | --- | --- | --- | --- | --- | --- | --- | --- | --- | --- | --- | --- | --- | --- |
|  |  |  |  |  | **ALS** | | |  | **Epilepsy** | | |  | **Meta-analysis** | | | |
| **Chr** | **bp** | **SNP** | **Risk allele** |  | **OR** | **se** | **p** |  | **OR** | **se** | **p** |  | **OR** | **se** | **p** | **Direction** |
| **ALS-All epilepsy** | | | | | | | | | | | | | | | | |
| ***1*** | ***39970928*** | ***rs61779331*** | ***A*** |  | ***1.01*** | ***4.6 x 10^-3^*** | ***2.3 x 10^-2^*** |  | ***1.02*** | ***4.0 x 10^-3^*** | ***8.7 x 10^-8^*** |  | ***1.02*** | ***3.0 x 10^-3^*** | ***3.2 x 10^-8^*** | ***++*** |
| 9 | 27545467 | rs812858 | T |  | 1.05 | 4.6 x 10^-3^ | 1.2 x 10^-24^ |  | 1.00 | 4.1 x 10^-3^ | 4.7 x 10^-1^ |  | 1.02 | 3.1 x 10^-3^ | 1.8 x 10^-13^ | ++ |
|  |  |  |  |  |  |  |  |  |  |  |  |  |  |  |  |  |
| **ALS-Focal epilepsy** | | | | | | | | | | | | | | | | |
| 9 | 27545467 | rs812858 | T |  | 1.05 | 4.6 x 10^-3^ | 1.2 x 10^-24^ |  | 1.00 | 4.1 x 10^-3^ | 3.1 x 10^-1^ |  | 1.02 | 3.1 x 10^-3^ | 3.2 x 10^-14^ | ++ |
|  |  |  |  |  |  |  |  |  |  |  |  |  |  |  |  |  |
| **ALS-Generalized epilepsy** | | | | | | | | | | | | | | | | |
| 2 | 58042241 | rs1402398 | G |  | 1.00 | 4.0 x 10^-3^ | 0.54 |  | 1.02 | 2.7 x 10^-3^ | 6.0 x 10^-11^ |  | 1.01 | 2.3 x 10^-3^ | 8.0 x 10^-9^ | ++ |
| 4 | 31148846 | rs1463849 | G |  | 1.00 | 4.0 x 10^-3^ | 0.27 |  | 1.02 | 2.7 x 10^-3^ | 9.3 x 10^-10^ |  | 1.01 | 2.3 x 10^-3^ | 1.5 x 10^-8^ | ++ |
| 9 | 27545960 | rs700791 | A |  | 1.05 | 4.6 x 10^-3^ | 1.2 x 10^-24^ |  | 1.00 | 3.3 x 10^-3^ | 5.2 x 10^-1^ |  | 1.02 | 2.7 x 10^-3^ | 1.1 x 10^-10^ | ++ |

| **B. Sign-independent meta-analysis** | | | | | | | | |
| --- | --- | --- | --- | --- | --- | --- | --- | --- |
|  |  |  |  |  |  | **Meta-analysis** | | |
| **Chr** | **Bp** | **SNP** | **Alleles** | **p ALS** | **p Epilepsy** | **OR** | **se** | **p** |
| **ALS-All epilepsy** | | | | | | | | |
| 1 | 39970928 | rs61779331 | A/C | 2.3 x 10^-2^ | 8.7 x 10^-8^ | 1.02 | 3.00 x 10^-3^ | 3.19 x 10^-8^ |
| 2 | 166998767 | rs6432877 | C/G | 8.0 x 10^-2^ | 8.9 x 10^-14^ | 1.02 | 3.00 x 10^-3^ | 1.14 x 10^-11^ |
| 9 | 27563755 | rs2484319 | A/C | 1.9 x 10^-26^ | 0.57 | 1.02 | 3.00 x 10^-3^ | 9.40 x 10^-14^ |
|  |  |  |  |  |  |  |  |  |
| **ALS-Focal epilepsy** | |  |  |  |  |  |  |  |
| 2 | 166998767 | rs6432877 | C/G | 8.0 x 10^-2^ | 8.9 x 10^-14^ | 1.02 | 3.00 x 10^-3^ | 4.46 x 10^-8^ |
| 9 | 27545467 | rs812858 | C/T | 1.2 x 10^-24^ | 0.31 | 1.02 | 3.10 x 10^-3^ | 3.15 x 10^-14^ |
|  |  |  |  |  |  |  |  |  |
| **ALS-Generalized epilepsy** | | | | | | | | |
| 2 | 58042241 | rs1402398 | A/G | 0.54 | 6.0 x 10^-11^ | 1.01 | 2.30 x 10^-3^ | 7.94 x 10^-9^ |
| 4 | 31148846 | rs1463849 | A/G | 0.27 | 9.3 x 10^-10^ | 1.01 | 2.30 x 10^-3^ | 1.48 x 10^-8^ |
| ***6*** | ***128332084*** | ***rs13219424*** | ***T/C*** | ***7.3 x 10^-3^*** | ***7.9 x 10^-9^*** | ***1.02*** | ***2.40 x 10^-3^*** | ***3.50 x 10^-10^*** |
| 9 | 27497988 | rs10967965 | A/T | 8.3 x 10^-22^ | 0.28 | 1.02 | 2.70 x 10^-3^ | 1.08 x 10^-10^ |

Table columns: Chr: chromosome; bp: basepair position (hg19/GRCh37); SNP: variant rs-number; Risk allele: disease-associated allele; OR: odds ratio of SNP in ALS, epilepsy and meta-analysis; se: standard error of SNP effect estimate in ALS, epilepsy and meta-analysis; p: association p-value of SNP in ALS, epilepsy and meta-analysis; Direction: direction of effect in ALS and epilepsy. Pleiotropic loci are highlighted.

**Consortium Members**

**Project MinE ALS GWAS consortium members**

Wouter van Rheenen^1^, Aleksey Shatunov^2^, Russell L. McLaughlin^3^, Rick A.A. van der Spek^1^, Alfredo Iacoangeli^2^, Kevin P. Kenna^1^, Kristel R. van Eijk^1^, Nicola Ticozzi^4, 5^, Boris Rogelj^6,7^, Katarina Vrabec^8^, Metka Ravnik-Glavač^8,9^, Blaž Koritnik^10^, Janez Zidar^10^, Lea Leonardis^10^, Leja Dolenc Grošelj^10^, Stéphanie Millecamps^11^, François Salachas^11, 12, 13^, Vincent Meininger^14,15^, Mamede de Carvalho16,17, Susana Pinto16, Marta Gromicho16, Ana Pronto-Laborinho16, Jesus S. Mora^18^, Ricardo Rojas-García^19^, Meraida Polak^21,22^, Siddharthan Chandran^23,24^, Shuna Colville^23^, Robert Swingler^23^, Karen E. Morrison^25^, Pamela J. Shaw^26^, John Hardy^27^, Richard W. Orrell^28^, Alan Pittman^27, 29^, Katie Sidle^28^, Pietro Fratta^30^, Andrea Malaspina^31, 32^, Simon Topp^2^, Susanne Petri^33^, Susanna Abdulla^34^, Carsten Drepper^35^, Michael Sendtner^35^, Thomas Meyer^36^, Roel A. Ophoff^37, 38^, Kim A. Staats^38^, Martina Wiedau-Pazos^39^, Catherine Lomen-Hoerth^40^, Vivianna M. Van Deerlin^41^, John Q. Trojanowski^41^, Lauren Elman^42^, Leo McCluskey^42^, A. Nazli Basak^43^, Thomas Meitinger^44^, Peter Lichtner^44^, Milena Blagojevic-Radivojkov^44^, Christian R. Andres^45^, Gilbert Bensimon^46, 47, 48^, Bernhard Landwehrmeyer^49^, Alexis Brice^50, 51, 52, 53, 54^, Christine A.M. Payan^46, 48^, Safaa Saker-Delye^55^, Alexandra Dürr^56^, Nicholas W. Wood^57^, Lukas Tittmann^58^, Wolfgang Lieb^58^, Andre Franke^59^, Marcella Rietschel^60^, Sven Cichon^61, 62, 63, 64, 65^, Markus M. Nöthen^61, 62^, Philippe Amouyel^66^, Christophe Tzourio^67^, Jean-François Dartigues^67^, Andre G. Uitterlinden^68, 69^, Fernando Rivadeneira^68, 69^, Karol Estrada^68^, Albert Hofman^69, 70^, Charles Curtis^71, 72^, Anneke J. van der Kooi^73^, Markus Weber^74^, Christopher E. Shaw^2^, Bradley N. Smith^2^, Daisy Sproviero^75^, Cristina Cereda^75^, Mauro Ceroni^76^, Luca Diamanti^76^, Roberto Del Bo^77^, Stefania Corti^77^, Giacomo P. Comi^77^, Sandra D'Alfonso^78^, Lucia Corrado^78^, Cinzia Bertolin^79^, Gianni Sorarù^79^, Letizia Mazzini^80^, Viviana Pensato^81^, Cinzia Gellera^81^, Cinzia Tiloca^4^, Antonia Ratti^4,5^, Andrea Calvo^82,83^, Cristina Moglia^82,83^, Maura Brunetti^82,83^, Simona Arcuti^84^, Rosa Capozzo^84^, Chiara Zecca^84^, Christian Lunetta^85^, Silvana Penco^86^, Nilo Riva^87^, Alessandro Padovani^88^, Massimiliano Filosto^89^, PARALS registry^90^, SLALOM group^90^, SLAP registry^90^, SLAGEN Consortium^90^, NNIPPS Study Group^90^, Ian Blair^91^, Garth A. Nicholson^91,92^, Dominic B. Rowe^91^, Roger Pamphlett^93^, Matthew C. Kiernan^94^, Julian Grosskreutz^95^, Otto W. Witte^95^, Robert Steinbach^95^, Tino Prell^95^, Beatrice Stubendorff^95^, Ingo Kurth^96, 97^, Christian A. Hübner^96^, P. Nigel Leigh^98^, Federico Casale^82^, Adriano Chio^82, 83^, Ettore Beghi^99^, Elisabetta Pupillo^99^, Rosanna Tortelli^84^, Giancarlo Logroscino^100, 101^, John Powell^2^, Albert C. Ludolph^49^, Jochen H. Weishaupt^49^, Wim Robberecht^102, 103, 104^, Philip Van Damme^102, 103, 104^, Robert H. Brown^105^, Jonathan D. Glass^21, 22^, John E. Landers^105^, Orla Hardiman^106, 107^, Peter M. Andersen^49, 108^, Philippe Corcia^45, 109, 110^, Patrick Vourc'h^45^, Vincenzo Silani^4,5^, Michael A. van Es^1^, R. Jeroen Pasterkamp^1^, Cathryn M. Lewis^71, 111^, Gerome Breen^71, 72^, Ammar Al-Chalabi^2^, Leonard H. van den Berg^1^, Jan H. Veldink^1^

1. Department of Neurology, UMC Utrecht Brain Center, University Medical Center Utrecht, Utrecht University, Utrecht, The Netherlands.
2. Maurice Wohl Clinical Neuroscience Institute, King's College London, Department of Basic and Clinical Neuroscience, London, UK.
3. Population Genetics Laboratory, Smurfit Institute of Genetics, Trinity College Dublin, Dublin, Republic of Ireland.
4. Department of Neurology and Laboratory of Neuroscience, IRCCS Istituto Auxologico Italiano, Milano, Italy.
5. Department of Pathophysiology and Tranplantation, ‘Dino Ferrari’ Center, Università degli Studi di Milano, Milano, Italy.
6. Department of Biotechnology, Jožef Stefan Institute, Ljubljana, Slovenia.
7. Biomedical Research Institute BRIS, Ljubljana, Slovenia.
8. Department of Molecular Genetics, Institute of Pathology, Faculty of Medicine, University of Ljubljana, SI-1000 Ljubljana, Slovenia.
9. Institute of Biochemistry, Faculty of Medicine, University of Ljubljana, SI-1000 Ljubljana, Slovenia.
10. Ljubljana ALS Centre, Institute of Clinical Neurophysiology, University Medical Centre Ljubljana, SI-1000 Ljubljana, Slovenia.
11. Institut du Cerveau et de la Moelle épinière, Inserm U1127, CNRS UMR 7225, Sorbonne Universités, UPMC Univ Paris 06 UMRS1127, Paris, France.
12. Centre de Référence Maladies Rares SLA Ile de France, Département de Neurologie, Hôpital de la Pitié-Salpêtrière, Paris, France.
13. GRC-UPMC SLA et maladies du Motoneurone, France.
14. Ramsay Generale de Santé, Hopital Peupliers, Paris, France.
15. Réseau SLA Ile de France.
16. Instituto de Fisiologia, Instituto de Medicina Molecular,Faculdade de Medicina, Universidade de Lisboa, Lisbon, Portugal
17. Department of Neurosciences, Hospital de Santa Maria-CHLN, Lisbon, Portugal.
18. Hospital San Rafael, Madrid, Spain
19. Neurology Department, Hospital de la Santa Creu i Sant Pau de Barcelona, Autonomous University of Barcelona, Barcelona, Spain.
20. Centro de Investigación en red en Enfermedades Raras (CIBERER), Spain.
21. Department Neurology, Emory University School of Medicine, Atlanta, GA, USA.
22. Emory ALS Center, Emory University School of Medicine, Atlanta, GA, USA.
23. Euan MacDonald Centre for Motor Neurone Disease Research, Edinburgh, UK.
24. Centre for Neuroregeneration and Medical Research Council Centre for Regenerative Medicine, University of Edinburgh, Edinburgh, UK.
25. Faculty of Medicine, University of Southampton, Southampton, UK.
26. Sheffield Institute for Translational Neuroscience (SITraN), University of Sheffield, Sheffield, UK.
27. Department of Molecular Neuroscience, Institute of Neurology, University College London, UK.
28. Department of Clinical Neuroscience, Institute of Neurology, University College London, UK.
29. Reta Lila Weston Institute, Institute of Neurology, University College London, UK.
30. Sobell Department of Motor Neuroscience and Movement Disorders, Institute of Neurology, University College London.
31. Centre for Neuroscience and Trauma, Blizard Institute, Queen Mary University of London, London, UK.
32. North-East London and Essex Regional Motor Neuron Disease Care Centre, London, UK.
33. Department of Neurology, Hannover Medical School, Hannover, Germany.
34. Department of Neurology, Otto-von-Guericke University Magdeburg, Magdeburg, Germany.
35. Institute of Clinical Neurobiology, University Hospital Wuerzburg, Germany.
36. Charité – Universitätsmedizin, Berlin, Germany.
37. Department of Human Genetics, David Geffen School of Medicine, University of California, Los Angeles, CA, USA.
38. Center for Neurobehavioral Genetics, Semel Institute for Neuroscience and Human Behavior, University of California, Los Angeles, CA, USA.
39. Department of Neurology, David Geffen School of Medicine, University of California, Los Angeles, CA, USA.
40. Department of Neurology, University of California, San Francisco, CA, USA.
41. Center for Neurodegenerative Disease Research, Perelman School of Medicine at the University of Pennsylvania, Philadelphia, PA, USA.
42. Department of Neurology, Perelman School of Medicine at the University of Pennsylvania, PA USA.
43. Koç University Medical School, Istanbul, Turkey.
44. Institute of Human Genetics, Helmholtz Zentrum München, Neuherberg, Germany.
45. Centre SLA, CHRU de Tours, Tours, France; UMR 1253, iBrain, Université de Tours, Inserm, Tours, France.
46. APHP, Département de Pharmacologie Clinique, Hôpital de la Pitié-Salpêtrière, France.
47. Université Pierre & Marie Curie, Pharmacologie, Paris VI, Paris, France.
48. BESPIM, CHU-Nîmes, Nîmes, France.
49. Department of Neurology, Ulm University, Ulm, Germany.
50. INSERM U 1127, Hôpital de la Pitié-Salpêtrière, 75013 Paris, France.
51. CNRS UMR 7225, Hôpital de la Pitié-Salpêtrière, 75013 Paris, France.
52. Sorbonne Universités, Université Pierre et Marie Curie Paris 06 UMRS 1127, Hôpital de la Pitié-Salpêtrière, 75013 Paris, France.
53. Institut du Cerveau et de la Moelle épinière, Hôpital de la Pitié-Salpêtrière, 75013 Paris, France.
54. APHP, Département de Génétique, Hôpital de la Pitié-Salpêtrière, 75013 Paris, France.
55. Genethon, CNRS UMR 8587 Evry, France.
56. Department of Medical Genetics, l’Institut du Cerveau et de la Moelle Épinière, Hoptial Salpêtrière, 75013 Paris, France.
57. Department of Neurogenetics, Institute of Neurology, University College London, UK.
58. PopGen Biobank and Institute of Epidemiology, Christian Albrechts-University Kiel, Kiel, Germany.
59. Institute of Clinical Molecular Biology, Kiel University, Kiel, Germany.
60. Department of Genetic Epidemiology in Psychiatry, Central Institute of Mental Health, Faculty of Medicine Mannheim, University of Heidelberg, Germany
61. Institute of Human Genetics, University of Bonn, Bonn, Germany.
62. Department of Genomics, Life and Brain Center, Bonn, Germany.
63. Division of Medical Genetics, University Hospital Basel, University of Basel, Basel, Switzerland.
64. Department of Biomedicine, University of Basel, Basel, Switzerland.
65. Institute of Neuroscience and Medicine INM-1, Research Center Juelich, Juelich, Germany.
66. University of Lille, Inserm, CHU Lille, Institut Pasteur de Lille, U1167 - RID-AGE - Risk Factor and molecular determinants of aging diseases, Labex Distalz, F-59000 Lille, France.
67. Bordeaux University, ISPED, Centre INSERM U1219-Epidemiologie-Biostatistique & CIC-1401, CHU de Bordeaux, Pole de Sante Publique, Bordeaux, France.
68. Department of Internal Medicine, Genetics Laboratory, Erasmus Medical Center Rotterdam, Rotterdam, The Netherlands.
69. Department of Epidemiology, Erasmus Medical Center Rotterdam, Rotterdam, The Netherlands.
70. Department of Epidemiology, Harvard T.H. Chan School of Public Health, Boston, MA, USA.
71. Social, Genetic & Developmental Psychiatry Centre, Institute of Psychiatry, Psychology & Neuroscience, King's College London, London, UK.
72. NIHR Maudsley Biomedical Research Centre, Maudsley Hospital and Institute of Psychiatry, Psychology & Neuroscience, King's College London, London, UK.
73. Amsterdam UMC, department of Neurology, University of Amsterdam, Neuroscience, Amsterdam
74. Neuromuscular Diseases Unit/ALS Clinic, Kantonsspital St. Gallen, 9007, St. Gallen, Switzerland.
75. Genomic and post-Genomic Center, IRCCS Mondino Foundation, Pavia, Italy.
76. General Neurology, IRCCS Mondino Foundation, Pavia, Italy
77. Neurologic Unit, IRCCS Foundation Ca’ Granda Ospedale Maggiore Policlinico, Milan, Italy.
78. Department of Health Sciences, Interdisciplinary Research Center of Autoimmune Diseases, UPO, Università del Piemonte Orientale, Novara, Italy.
79. Department of Neurosciences, University of Padova, Padova, Italy.
80. Department of Neurology, University of Eastern Piedmont, Novara, Italy.
81. Unit of Genetics of Neurodegenerative and Metabolic Diseases, Fondazione IRCCS Istituto Neurologico ‘Carlo Besta’, Milano, Italy.
82. “Rita Levi Montalcini” Department of Neuroscience, ALS Centre, University of Torino, Turin, Italy.
83. Azienda Ospedaliera Città della Salute e della Scienza, Torino, Italy.
84. Department of Clinical research in Neurology, University of Bari "A. Moro", at Pia Fondazione "Card. G. Panico", Tricase (LE), Italy.
85. NEMO Clinical Center, Serena Onlus Foundation, Niguarda Ca' Granda Hostipal, Milan, Italy.
86. Medical Genetics Unit, Department of Laboratory Medicine, Niguarda Ca' Granda Hospital, Milan, Italy.
87. Department of Neurology, Institute of Experimental Neurology (INSPE), Division of Neuroscience, San Raffaele Scientific Institute, Milan, Italy.
88. Neurology Unit, Department of Clinical and Experimental Sciences, University of Brescia, Italy.
89. Neurology Unit, Department of Neuroscience and Vision, Spedali Civili Hospital, Brescia, Italy.
90. A list of members and affiliations appears in the Supplementary Note.
91. Department of Biomedical Sciences, Faculty of Medicine and Health Sciences, Macquarie University, Sydney, New South Wales, Australia.
92. University of Sydney, ANZAC Research Institute, Concord Hospital, Sydney, New South Wales, Australia.
93. The Stacey MND Laboratory, Department of Pathology, The University of Sydney, Sydney, New South Wales, Australia.
94. Brain and Mind Centre, The University of Sydney, New South Wales 2050, Australia.
95. Hans-Berger Department of Neurology, Jena University Hospital, Jena, Germany.
96. Institute of Human Genetics, Jena University Hospital, Jena, Germany.
97. Institute of Human Genetics, Medical Faculty, RWTH Aachen University, Aachen, Germany
98. Department of Neurology, Brighton and Sussex Medical School Trafford Centre for Biomedical Research, University of Sussex, Falmer, East Sussex, UK.
99. Laboratory of Neurological Diseases, Department of Neuroscience, IRCCS Istituto di Ricerche Farmacologiche Mario Negri, Milano, Italy.
100. Department of Basic Medical Sciences, Neuroscience and Sense Organs, University of Bari 'Aldo Moro', Bari, Italy.
101. Unit of Neurodegenerative Diseases, Department of Clinical Research in Neurology, University of Bari 'Aldo Moro', at Pia Fondazione Cardinale G. Panico, Tricase, Lecce, Italy.
102. KU Leuven - University of Leuven, Department of Neurosciences
103. VIB, Center for Brain & Disease Research, Laboratory of Neurobiology, Leuven, Belgium.
104. University Hospitals Leuven, Department of Neurology, Leuven, Belgium.
105. Department of Neurology, University of Massachusetts Medical School, Worcester, MA, USA.
106. Academic Unit of Neurology, Trinity College Dublin, Trinity Biomedical Sciences Institute, Dublin, Republic of Ireland.
107. Department of Neurology, Beaumont Hospital, Dublin, Republic of Ireland.
108. Department of Pharmacology and Clinical Neurosience, Umeå University, Umeå, Sweden.
109. Federation des Centres SLA Tours and Limoges, LITORALS, Tours, France.
110. INSERM U930, Université François Rabelais, Tours, France.
111. Department of Medical and Molecular Genetics, King's College London, London, UK.

*Italian Consortium for the Genetics of ALS (SLAGEN) members*

Daniela Calini, Isabella Fogh, Antonia Ratti, Vincenzo Silani, Nicola Ticozzi, Cinzia Tiloca, Barbara Castellotti, Cinzia Gellera, Viviana Pensato, Franco Taroni, Cristina Cereda, Mauro Ceroni, Stella Gagliardi, Giacomo Comi, Stefania Corti, Roberto Del Bo, Lucia Corrado, Sandra D’Alfonso, Letizia Mazzini, Elena Pegoraro, Giorgia Querin and Gianni Sorarù

*Registro Lombardo Sclerosi Laterale Amyotrofica (SLALOM) group members*

Francesca Gerardi, Fabrizio Rinaldi, Maria Sofia Cotelli, Luca Chiveri, Maria Cristina Guaita, Patrizia Perrone, Giancarlo Comi, Carlo Ferrarese, Lucio Tremolizzo, Marialuisa Delodovici and Giorgio Bono

*Piemonte and Valle d'Aosta Registry for Amyotrophic Lateral Sclerosis (PARALS) group members*

Stefania Cammarosano, Antonio Canosa, Dario Cocito, Leonardo Lopiano, Luca Durelli, Bruno Ferrero, Antonio Bertolotto, Alessandro Mauro, Luca Pradotto, Roberto Cantello, Enrica Bersano, Dario Giobbe, Maurizio Gionco, Daniela Leotta, Lucia Appendino, Roberto Cavallo, Enrico Odddenino, Claudio Geda, Fabio Poglio, Paola Santimaria, Umberto Massazza, Antonio Villani, Roberto Conti, Fabrizio Pisano, Mario Palermo, Franco Vergnano, Paolo Provera, Maria Teresa Penza, Marco Aguggia, Nicoletta Di Vito, Piero Meineri, Ilaria Pastore, Paolo Ghiglione, Danilo Seliak, Nicola Launaro, Giovanni Astegiano and Bottacchi Edo

*Sclerosi Laterale Amyotrofica-Puglia (SLAP) registry members*

Isabella Laura Simone, Stefano Zoccolella, Michele Zarrelli and Franco Apollo

*Neuroprotection and Natural History in Parkinson Plus Syndromes (NNIPPS) Study group members*

William Camu, Jean Sebastien Hulot, Francois Viallet, Philippe Couratier, David Maltete, Christine Tranchant, Marie Vidailhet.

**International League Against Epilepsy Consortium on Complex Epilepsies members**

*Members listed in alphabetical order:*

Bassel Abou-Khalil^1^, Pauls Auce^2, 3^, Andreja Avbersek^4^, Melanie Bahlo^5-7^, David J Balding^8, 9^, Thomas Bast^10, 11^, Larry Baum^12-14^, Albert J Becker^15^, Felicitas Becker^16, 17^ Bianca Berghuis^18^, Samuel F Berkovic^19^, Katja E Boysen^19^, Jonathan P Bradfield^20, 21^, Lawrence C Brody^22^, Russell J Buono^20, 23, 24^, Ellen Campbell^25^, Gregory D Cascino^26^, Claudia B Catarino^4^, Gianpiero L Cavalleri^27, 28^, Stacey S Cherny^13, 29^, Krishna Chinthapalli^4^, Alison J Coffey^30^, Alastair Compston^31^, Antonietta Coppola^32, 33^, Patrick Cossette^34^, John J Craig^35^, Gerrit-Jan de Haan^36^, Peter De Jonghe^37, 38^, Carolien G F de Kovel^39^, Norman Delanty^27, 28, 40^, Chantal Depondt^41^, Orrin Devinsky^42^, Dennis J Dlugos^43^, Colin P Doherty^28, 44^, Christian E Elger^45^, Johan G Eriksson^46^, Thomas N Ferraro^23, 47^, Martha Feucht^48^, Ben Francis^49^, Andre Franke^50^, Jacqueline A French^51^, Saskia Freytag^5^, Verena Gaus^52^, Eric B Geller^53^, Christian Gieger^54, 55^, Tracy Glauser^56^, Simon Glynn^57^, David B Goldstein^58, 59^, Hongsheng Gui^13^, Youling Guo^13^, Kevin F Haas^1^, Hakon Hakonarson^20, 60^, Kerstin Hallmann^45, 61^, Sheryl Haut^62^, Erin L Heinzen^58, 59^, Ingo Helbig^43, 63^, Christian Hengsbach^16^, Helle Hjalgrim^64, 65^, Michele Iacomino^33^, Andrés Ingason^66^, Jennifer Jamnadas-Khoda^4, 67^, Michael R Johnson^68^, Reetta Kälviäinen^69, 70^, Anne-Mari Kantanen^69^, Dalia Kasperavičiūte^4^, Dorothee Kasteleijn-Nolst Trenite^39^, Heidi E Kirsch^71^, Robert C Knowlton^72^, Bobby P C Koeleman^39^, Roland Krause^73^, Martin Krenn^74^, Wolfram S Kunz^45^, Ruben Kuzniecky^75^, Patrick Kwan^12, 76, 77^, Dennis Lal^78^, Yu-Lung Lau^79^, Anna-Elina Lehesjoki^80^, Holger Lerche^16^, Costin Leu^4, 78, 81^, Wolfgang Lieb^82^, Dick Lindhout^36, 39^, Warren D Lo^83^, Iscia Lopes-Cendes^84, 85^, Daniel H Lowenstein^71^, Alberto Malovini^86^, Anthony G Marson^2^, Thomas Mayer^87^, Mark McCormack^27^, James L Mills^88^, Nasir Mirza^2^, Martina Moerzinger^48^, Rikke S Møller^64, 65, 89^, Anne M Molloy^90^, Hiltrud Muhle^63^, Mark Newton^91^, Ping-Wing Ng^92^, Markus M Nöthen^93^, Peter Nürnberg^94^, Terence J O’Brien^76, 77^, Karen L Oliver^19^, Aarno Palotie^95, 96^, Faith Pangilinan^22^, Sarah Peter^73^, Slavé Petrovski^76, 97^, Annapurna Poduri^98^, Michael Privitera^99^, Rodney Radtke^100^, Sarah Rau^16^, Philipp S Reif^101, 102^, Eva M Reinthaler^74^, Felix Rosenow^101, 102^, Josemir W Sander^4, 36, 103^, Thomas Sander^52, 94^, Theresa Scattergood^104^, Steven C Schachter^105^, Christoph J Schankin^106^, Ingrid E Scheffer^19, 107^, Bettina Schmitz^52^, Susanne Schoch^15^, Pak C Sham^13^, Jerry J Shih^108^, Graeme J Sills^2^, Sanjay M Sisodiya^4, 103^, Lisa Slattery^109^, Alexander Smith^78^, David F Smith^3^, Michael C Smith^110^, Philip E Smith^111^, Anja C M Sonsma^39^, Doug Speed^8, 112^, Michael R Sperling^113^, Bernhard J Steinhoff^10^, Ulrich Stephani^63^, Remi Stevelink^39^, Konstantin Strauch^114, 115^, Pasquale Striano^116^, Hans Stroink^117^, Rainer Surges^45^, K Meng Tan^76^, Liu Lin Thio^118^, G Neil Thomas^119^, Marian Todaro^76^, Rossana Tozzi^120^, Maria S Vari^116^, Eileen P G Vining^121^, Frank Visscher^122^, Sarah von Spiczak^63^, Nicole M Walley^58, 123^, Yvonne G Weber^16,126^, Zhi Wei^124^, Judith Weisenberg^118^, Christopher D Whelan^27^, Peter Widdess-Walsh^53^, Markus Wolff^125^, Stefan Wolking^16^, Wanling Yang^79^, Federico Zara^33^, Fritz Zimprich^74^

1. Vanderbilt University Medical Center, Nashville, TN 37232, USA.
2. Department of Molecular and Clinical Pharmacology, University of Liverpool, Liverpool L69 3GL, UK.
3. The Walton Centre NHS Foundation Trust, Liverpool L9 7LJ, UK.
4. Department of Clinical and Experimental Epilepsy, UCL Institute of Neurology, Queen Square, London WC1N 3BG, UK.
5. Population Health and Immunity Divison, The Walter and Eliza Hall Institute of Medical Research, Parkville 3052, Australia.
6. Department of Biology, University of Melbourne, Parkville 3010, Australia.
7. School of Mathematics and Statistics, University of Melbourne, Parkville 3010, Australia.
8. UCL Genetics Institute, University College London, London WC1E 6BT, UK.
9. Melbourne Integrative Genomics, University of Melbourne, Parkville 3052, Australia.
10. Epilepsy Center Kork, Kehl-Kork 77694, Germany.
11. Medical Faculty of the University of Freiburg, Freiburg 79085, Germany.
12. Centre for Genomic Sciences, The University of Hong Kong, Hong Kong.
13. Department of Psychiatry, The University of Hong Kong, Hong Kong.
14. The State Key Laboratory of Brain and Cognitive Sciences, University of Hong Kong, Hong Kong, China.
15. Section for Translational Epilepsy Research, Department of Neuropathology, University of Bonn Medical Center, Bonn 53105, Germany.
16. Department of Neurology and Epileptology, Hertie Institute for Clinical Brain Research, University of Tübingen, Tübingen 72076, Germany.
17. Department of Neurology, University of Ulm, Ulm 89081, Germany.
18. Stichting Epilepsie Instellingen Nederland (SEIN), Zwolle 8025 BV, The Netherlands.
19. Epilepsy Research Centre, University of Melbourne, Austin Health, Heidelberg 3084, Australia.
20. Center for Applied Genomics, The Children's Hospital of Philadelphia, Philadelphia, PA 19104, USA.
21. Quantinuum Research LLC, San Diego, CA 92101, USA.
22. National Human Genome Research Institute, National Institutes of Health, Bethesda, MD 20892, USA.
23. Department of Biomedical Sciences, Cooper Medical School of Rowan University Camden, NJ 08103, USA.
24. Department of Neurology, Thomas Jefferson University Hospital, Philadelphia, PA 19107, USA.
25. Belfast Health and Social Care Trust, Belfast BT9 7AB, UK.
26. Division of Epilepsy, Department of Neurology, Mayo Clinic, Rochester, MN 55902, USA.
27. Department of Molecular and Cellular Therapeutics, The Royal College of Surgeons in Ireland, Dublin 2, Ireland.
28. The FutureNeuro Research Centre, Dublin 2, Ireland.
29. Department of Epidemiology and Preventive Medicine, School of Public Health, Sackler Faculty of Medicine, Tel Aviv University, Tel Aviv 6997801, Israel.
30. The Wellcome Trust Sanger Institute, Hinxton, Cambridge CB10 1SA, UK.
31. Department of Clinical Neurosciences, Cambridge Biomedical Campus, Cambridge CB2 0SL, UK.
32. Department of Neuroscience, Reproductive and Odontostomatological Sciences, University Federico II, Naples 80138, Italy.
33. Laboratory of Neurogenetics and Neurosciences, Institute G. Gaslini, Genova 16148, Italy.
34. Department of Neurosciences, Université de Montréal, Montréal, CA 26758, Canada.
35. Department of Neurology, Royal Victoria Hospital, Belfast Health and Social Care Trust, Grosvenor Road, Belfast BT12 6BA, UK.
36. Stichting Epilepsie Instellingen Nederland (SEIN), Heemstede 2103 SW, The Netherlands.
37. Neurogenetics Group, Center for Molecular Neurology, VIB and Laboratory of Neurogenetics, Institute Born-Bunge, University of Antwerp, Antwerp 2610, Belgium.
38. Department of Neurology, Antwerp University Hospital, Edegem 2650, Belgium.
39. Department of Genetics, University Medical Center Utrecht, Utrecht 3584 CX, The Netherlands.
40. Division of Neurology, Beaumont Hospital, Dublin D09 FT51, Ireland.
41. Department of Neurology, Hôpital Erasme, Université Libre de Bruxelles, Bruxelles 1070, Belgium.
42. Comprehensive Epilepsy Center, New York University School of Medicine, New York, NY 10016, USA.
43. Department of Neurology, The Children's Hospital of Philadelphia, Philadelphia, PA 19104, USA.
44. Neurology Department, St. James’s Hospital, Dublin D03 VX82, Ireland.
45. Department of Epileptology, University of Bonn Medical Centre, Bonn 53127, Germany.
46. Department of General Practice and Primary Health Care, University of Helsinki and Helsinki University Hospital, Helsinki 0014, Finland.
47. Department of Pharmacology and Psychiatry, University of Pennsylvania Perlman School of Medicine, Philadelphia, PA 19104, USA.
48. Department of Pediatrics and Neonatology, Medical University of Vienna, Vienna 1090, Austria.
49. Department of Biostatistics, University of Liverpool, Liverpool L69 3GL, UK.
50. Institute of Clinical Molecular Biology, Christian-Albrechts-University of Kiel, University Hospital Schleswig Holstein, Kiel 24105, Germany.
51. Department of Neurology, NYU School of Medicine, New York City, NY 10003, USA.
52. Department of Neurology, Charité Universitaetsmedizin Berlin, Campus Virchow-Clinic, Berlin 13353, Germany.
53. Institute of Neurology and Neurosurgery at St. Barnabas, Livingston, NJ 07039, USA.
54. Research Unit of Molecular Epidemiology, Helmholtz Zentrum München - German Research Center for Environmental Health, Neuherberg D-85764, Germany.
55. Institute of Epidemiology, Helmholtz Zentrum München - German Research Center for Environmental Health, Neuherberg D-85764, Germany.
56. Comprehensive Epilepsy Center, Division of Neurology, Cincinnati Children's Hospital Medical Center, Cincinnati, OH 45229, USA.
57. Department of Neurology, University of Michigan, Ann Arbor, MI 48109, USA.
58. Center for Human Genome Variation, Duke University School of Medicine, Durham, NC 27710, USA.
59. Institute for Genomic Medicine, Columbia University Medical Center, New York, NY 10032, USA.
60. Division of Human Genetics, Department of Pediatrics, The Perelman School of Medicine, University of Pennsylvania, Philadelphia, PA 19104, USA.
61. Life and Brain Center, University of Bonn Medical Center, Bonn 53127, Germany.
62. Montefiore Medical Center, Bronx, NY 10467, USA.
63. Department of Neuropediatrics, University Medical Center Schleswig-Holstein (UKSH), Kiel 24105, Germany.
64. Danish Epilepsy Centre, Dianalund 4293, Denmark.
65. Institute of Regional Health Services Research, University of Southern Denmark, Odense 5000, Denmark.
66. deCODE genetics, Reykjavik IS-101, Iceland.
67. Department of Psychiatry and Applied Psychology, Institute of Mental Health University of Nottingham, Nottingham NG7 2TU, UK.
68. Faculty of Medicine, Imperial College London, London SW7 2AZ, UK.
69. Kuopio Epilepsy Center, Neurocenter, Kuopio University Hospital, Kuopio 70029, Finland.
70. Institute of Clinical Medicine, University of Eastern Finland, Kuopio 70029, Finland.
71. Department of Neurology, University of California, San Francisco, CA 94143, USA.
72. University of Alabama Birmingham, Department of Neurology, Birmingham, AL 35233, USA.
73. Luxembourg Centre for Systems Biomedicine, University of Luxembourg, Esch-sur-Alzette L-4362, Luxembourg.
74. Department of Neurology, Medical University of Vienna, Vienna 1090, Austria.
75. Department of Neurology, Zucker-Hofstra Northwell School of Medicine, NY 10075, USA.
76. Department of Medicine, University of Melbourne, Royal Melbourne Hospital, Parkville 3050, Australia.
77. Department of Neuroscience, Central Clinical School, Monash University, Melbourne 3004, Australia.
78. Stanley Center for Psychiatric Research, Broad Institute of Harvard and M.I.T., Cambridge, MA 02142, USA.
79. Department of Paediatrics and Adolescent Medicine, The University of Hong Kong, Hong Kong.
80. Folkhälsan Research Center and Medical Faculty, University of Helsinki, Helsinki 00290, Finland.
81. Genomic Medicine Institute, Lerner Research Institute, Cleveland Clinic, Cleveland, OH 44195, USA.
82. Institut für Epidemiologie Christian-Albrechts-Universität zu Kiel, Kiel 24105, Germany.
83. Department of Pediatrics and Neurology, Ohio State University and Nationwide Children's Hospital, Columbus, OH 43205, USA.
84. Department of Medical Genetics, School of Medical Sciences, University of Campinas (UNICAMP), Campinas, SP 13083-887, Brazil.
85. Brazilian Institute of Neuroscience and Neurotechnology (BRAINN), Campinas, SP 13083-970, Brazil.
86. Istituti Clinici Scientifici Maugeri, Pavia 27100, Italy.
87. Epilepsy Center Kleinwachau, Radeberg 01454, Germany.
88. Division of Intramural Population Health Research, Eunice Kennedy Shriver National Institute of Child Health and Human Development, National Institutes of Health, Bethesda, MD 20892, USA.
89. Wilhelm Johannsen Centre for Functional Genome Research, Copenhagen DK-2200, Denmark.
90. School of Medicine, Trinity College Dublin, Dublin 2, Ireland.
91. Department of Neurology, Austin Health, Heidelberg 3084, Australia.
92. United Christian Hospital, Hong Kong.
93. Institute of Human Genetics, University of Bonn Medical Center, Bonn 53127, Germany.
94. Cologne Center for Genomics, University of Cologne, Cologne 50931, Germany.
95. Institute for Molecular Medicine Finland (FIMM), University of Helsinki, Helsinki 0014, Finland.
96. The Broad Institute of M.I.T. and Harvard, Cambridge, MA 02142, USA.
97. AstraZeneca Centre for Genomics Research, Precision Medicine and Genomics, IMED Biotech Unit, AstraZeneca, Cambridge CB2 0AA, UK.
98. Department of Neurology, Boston Children's Hospital, Harvard Medical School, Boston, MA 02115, USA.
99. Department of Neurology, Neuroscience Institute, University of Cincinnati Medical Center, Cincinnati, OH 45220, USA.
100. Department of Neurology, Duke University School of Medicine, Durham, NC 27710, USA.
101. Epilepsy-Center Hessen, Department of Neurology, University Medical Center Giessen and Marburg, Marburg, Germany and Philipps-University Marburg, Marburg 35043, Germany.
102. Epilepsy Center Frankfurt Rhine-Main, Center of Neurology and Neurosurgery, Goethe University Frankfurt, Frankfurt 60528, Germany.
103. Chalfont Centre for Epilepsy, Chalfont-St-Peter, Buckinghamshire SL9 0RJ, UK.
104. Department of Endocrinology, Hospital of The University of Pennsylvania, Philadelphia, PA 19104, USA.
105. Departments of Neurology, Beth Israel Deaconess Medical Center, Massachusetts General Hospital, and Harvard Medical School, Boston, MA 02215, USA.
106. Department of Neurology, Inselspital, Bern University Hospital, University of Bern, Bern 3010, Switzerland.
107. Department of Neurology, Royal Children's Hospital, Parkville 3052, Australia.
108. Department of Neurosciences, University of California, San Diego, La Jolla, CA 92037, USA.
109. The Royal College of Surgeons in Ireland, Dublin D02 YN77, Ireland. .
110. Rush University Medical Center, Chicago, IL 60612, USA.
111. Department of Neurology, Alan Richens Epilepsy Unit, University Hospital of Wales, Cardiff CF14 4XW, UK.
112. Aarhus Institute of Advanced Studies (AIAS), Aarhus University, 8000 Aarhus, Denmark.
113. Department of Neurology and Comprehensive Epilepsy Center, Thomas Jefferson University, Philadelphia, PA 19107, USA.
114. Institute of Genetic Epidemiology, Helmholtz Zentrum München - German Research Center for Environmental Health, Neuherberg D-85764, Germany.
115. Chair of Genetic Epidemiology, IBE, Faculty of Medicine, LMU Munich 80539, Germany.
116. Pediatric Neurology and Muscular Diseases Unit, Department of Neurosciences, Rehabilitation, Ophthalmology, Genetics, Maternal and Child Health, G. Gaslini Institute, University of Genoa, Genova 16148, Italy.
117. CWZ Hospital, 6532 SZ Nijmegen, The Netherlands.
118. Department of Neurology, Washington University School of Medicine, St. Louis, MO 63110, USA.
119. Institute for Applied Health Research, University of Birmingham, Birmingham B15 2TT, UK.
120. C. Mondino National Neurological Institute, Pavia 27100, Italy.
121. Departments of Neurology and Pediatrics, The Johns Hopkins University School of Medicine, Baltimore, MD 21287, USA.
122. Department of Neurology, Admiraal De Ruyter Hospital, Goes 4462, The Netherlands.
123. Division of Medical Genetics, Department of Pediatrics, Duke University Medical Center, Durham, NC 27710, USA.
124. Department of Computer Science, New Jersey Institute of Technology, NJ 07102, USA.
125. Department of Pediatric Neurology and Developmental Medicine, University Children's Hospital, Tübingen 72076, Germany.
126. Department of Neurosurgery, University of Tübingen, Tübingen 72076, Germany.
